# Nuclear Aurora kinase A switches m^6^A reader YTHDC1 to enhance an oncogenic RNA splicing of tumor suppressor RBM4

**DOI:** 10.1038/s41392-022-00905-3

**Published:** 2022-04-01

**Authors:** SiSi Li, YangFan Qi, JiaChuan Yu, YuChao Hao, Bin He, MengJuan Zhang, ZhenWei Dai, TongHui Jiang, SuYi Li, Fang Huang, Ning Chen, Jing Wang, MengYing Yang, DaPeng Liang, Fan An, JinYao Zhao, WenJun Fan, YuJia Pan, ZiQian Deng, YuanYuan Luo, Tao Guo, Fei Peng, ZhiJie Hou, ChunLi Wang, FeiMeng Zheng, LingZhi Xu, Jie Xu, QingPing Wen, BiLian Jin, Yang Wang, Quentin Liu

**Affiliations:** 1 Institute of Cancer Stem Cell, Cancer Center, Dalian Medical University, Dalian, China; 2 State Key Laboratory of Oncology in South China, Collaborative Innovation Center for Cancer Medicine, Sun Yat-sen University, Guangzhou, China; 3 Department of Pathology, Harbin Medical University Cancer Hospital, Harbin, China; 4 Department of Anesthesiology, the First Affiliated Hospital of Dalian Medical University, Dalian, China; 5 Department of Oncology, the First Affiliated Hospital of Gannan Medical College, Ganzhou, China; 6 Department of Oncology, the First Affiliated Hospital of Dalian Medical University, Dalian, China; 7 Department of Thoracic surgery, the First Affiliated Hospital of Dalian Medical University, Dalian, China; 8 Department of Oncology, the Second Affiliated Hospital of Dalian Medical University, Dalian, China

**Keywords:** RNA splicing, Lung cancer

## Abstract

Aberrant RNA splicing produces alternative isoforms of genes to facilitate tumor progression, yet how this process is regulated by oncogenic signal remains largely unknown. Here, we unveil that non-canonical activation of nuclear AURKA promotes an oncogenic RNA splicing of tumor suppressor RBM4 directed by m^6^A reader YTHDC1 in lung cancer. Nuclear translocation of AURKA is a prerequisite for RNA aberrant splicing, specifically triggering RBM4 splicing from the full isoform (RBM4-FL) to the short isoform (RBM4-S) in a kinase-independent manner. RBM4-S functions as a tumor promoter by abolishing RBM4-FL-mediated inhibition of the activity of the SRSF1-mTORC1 signaling pathway. Mechanistically, AURKA disrupts the binding of SRSF3 to YTHDC1, resulting in the inhibition of RBM4-FL production induced by the m^6^A-YTHDC1-SRSF3 complex. In turn, AURKA recruits hnRNP K to YTHDC1, leading to an m^6^A-YTHDC1-hnRNP K-dependent exon skipping to produce RBM4-S. Importantly, the small molecules that block AURKA nuclear translocation, reverse the oncogenic splicing of RBM4 and significantly suppress lung tumor progression. Together, our study unveils a previously unappreciated role of nuclear AURKA in m^6^A reader YTHDC1-dependent oncogenic RNA splicing switch, providing a novel therapeutic route to target nuclear oncogenic events.

## Introduction

Splicing of mRNA precursors (pre-mRNA) that enables multiple transcripts from a single gene is the main reason for maintaining proteomic diversity.^1^ Alternative splicing (AS) is generally executed by *trans*-acting splicing factors, which bind to *cis*-elements that lie in both exons and introns to affect the usage of adjacent splice sites.^2^ Classical splicing factors include serine/arginine-rich proteins and heterogeneous nuclear ribonucleoproteins (hnRNPs) that promote or suppress splicing via binding to splicing enhancers or silencers in a context-dependent manner.^3^ Accumulating evidence demonstrates that splicing disorders are implicated in multiple human cancers, and contribute to tumorigenesis and tumor progression through the production of abnormal protein and gene isoforms.^4^ For example, downregulation of splicing regulator MBNL1 facilitates the splicing of exon 2 of cancer stemness related gene *MAP2K7* to generate stemness promoting subtype MAP2K7Δexon 2, thereby enhancing tumorigenic and stem/progenitor-like properties.^5^ Reduction of hnRNP E1 promotes the production of the lncRNA-PNUTS subtype of PNUTS, mediating epithelial-mesenchymal transformation (EMT) associated tumor progression.^6^ Therefore, the discovery of novel tumor-related abnormal splicing events and the exploration of the detailed mechanisms underlying deregulated splicing will provide effective strategies for treating tumors by correcting RNA aberrant splicing.

Recent studies demonstrate that RNA splicing is frequently under the control of N^6^-methyladenosine (m^6^A) modification of RNA.^7–9^ m^6^A methyltransferase complex consisting of METTL3, METTL14, and WTAP plays a critical role in regulating alternative splicing of genes involved in transcription and RNA processing via catalyzing m^6^A formation.^10^ The m^6^A demethylating enzyme FTO, however, changes the RNA-binding ability of splicing regulator SRSF2 to influence RNA splicing via removing m^6^A modification.^11^ Of note, the m^6^A reader YTHDC1 directly promotes exon inclusion through recruiting distinct splicing factors to m^6^A sites, thus playing an essential role in bridging interactions of *trans-* and *cis-* regulatory.^12^ YTHDC1-deficient oocytes exhibit extensive RNA splicing defects, which are rescued by introducing YTHDC1 wild-type rather than m^6^A-binding region mutant.^13^ Yet, how RNA splicing, especially tumor-related aberrant RNA splicing, is controlled by m^6^A and its readers remains to be elucidated.

Aurora kinase A (AURKA) plays an important oncogenic role in promoting tumor initiation and progression.^14^ We have previously identified that nuclear AURKA *trans*-activates *MYC* and Forkhead box subclass M1 (FOXM1) target genes expression, which is crucial for breast cancer stem cells (BCSCs) self-renewal.^15,16^ Almost recently, our study shows that AURKA increases DROSHA mRNA stability and subsequently enhances BCSCs phenotype.^17^ Now we discover that nuclear translocation of AURKA regulates m^6^A reader YTHDC1-mediated RNA splicing in a kinase-independent way, leading to an oncogenic splicing switch of tumor suppressor RBM4 in lung cancer. AURKA interferes with the YTHDC1-SRSF3 complex, thus inhibiting the production of tumor-suppressing isoform RBM4-FL. In turn, AURKA links the interaction between hnRNP K and YTHDC1, thereby enhancing the production of tumor-promoting isoform RBM4-S. AURKA nuclear translocation inhibitors reverse the oncogenic splicing of RBM4 and significantly suppress lung tumor progression. Together, our findings reveal that the m^6^A reader YTHDC1-directed RBM4 aberrant splicing is triggered by nuclear AURKA, providing novel opportunities for targeted therapy of lung cancer by blocking nuclear oncogenic signaling.

## Results

### A non-canonical function of nuclear AURKA in driving RBM4 aberrant RNA splicing

**Fig. 1:**
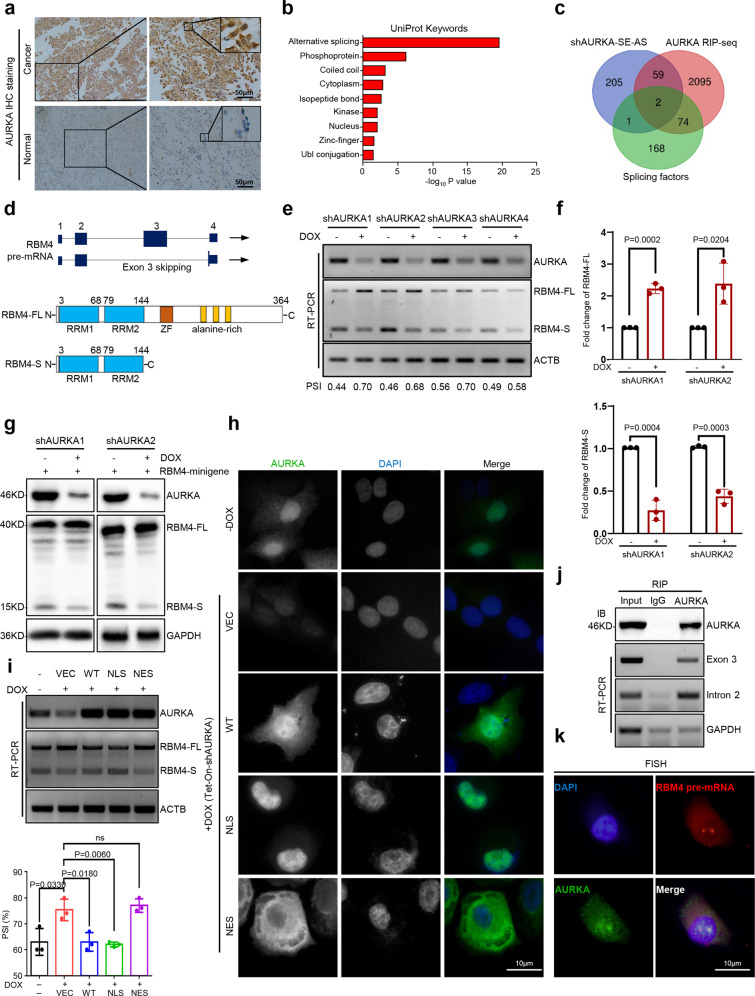
A non-canonical function of nuclear AURKA in driving RBM4 aberrant RNA splicing. **a** Representative immunohistochemistry (IHC) staining showing AURKA expression in non-small cell lung cancer (NSCLC) specimens and adjacent normal tissues. Right images show ×20 and ×40 magnification of region indicated by the black box in the corresponding image. Scale bar, 50 μm. **b** UniProt Keywords analysis of AURKA-regulated splicing events of SE type. −log_10_ *P*-values were plotted for each enriched functional classification. **c** Comparison of AURKA-regulated splicing events of SE type with AURKA RNA-immunoprecipitation sequencing (RIP-seq) data and one RNA splicing-related gene set. Two candidates, SRRM1 and RBM4. **d** Schematics of human RBM4 pre-mRNA and protein. The full-length RBM4 (RBM4-FL) contains 1–4 exons, whereas exon 3 of RBM4 can be skipped to generate a new short isoform RBM4-S. RBM4-S only contains an N-terminal RNA-binding domain. **e** Validation of RBM4 splicing change by semi-quantitative RT-PCR in DOX-induced AURKA knockdown NCI-H460 cells. PSI (Percent Spliced In) = RBM4-FL/RBM4-FL + RBM4-S. DOX, 2 μg/ml. **f** RT-qPCR assay was conducted to detect RBM4-FL and RBM4-S mRNA expression levels in DOX-induced AURKA knockdown NCI-H460 cells. **g** RBM4 splicing reporter was overexpressed in control and AURKA knockdown NCI-H460 cells. Western blot was conducted to assay for the splicing change of RBM4. **h** Empty vector (VEC), AURKA-WT (wild-type), AURKA-NLS (nuclear-localized sequence), and AURKA-NES (nuclear export sequence) were transfected into endogenous AURKA depleted NCI-H460 cells. The localization of AURKA (green) was detected by immunofluorescence (IF) assay with anti-AURKA antibody. The nuclei were stained with DAPI (blue). Scale bar, 10 μm. **i** RBM4 splicing change in VEC, AURKA-WT, AURKA-NLS, and AURKA-NES reconstitution NCI-H460-shAURKA cells was measured by RT-PCR. **j** Binding of RBM4 pre-mRNA with AURKA protein was detected by RIP assay in NCI-H460 cells. **k** Colocalization of RBM4 pre-mRNA (red) with AURKA protein (green) was detected by RNA fluorescence in situ hybridization (FISH) assay in NCI-H460 cells. The nucleus was stained with DAPI (blue). Scale bar, 10 μm. Data are shown as means ± SD. *P*-values were calculated with two-tailed unpaired Student’s *t*-test and *P* < 0.05 is considered statistically significant

To further investigate the tumor-promoting mechanism of AURKA, we firstly examined the localization and expression of AURKA protein in lung cancer and adjacent normal tissues. Immunohistochemistry (IHC) staining showed that AURKA was highly accumulated in the nuclei of cancer tissues compared to adjacent tissues (Fig. 1a). Consistently, AURKA expression in lung cancer cell lines was significantly higher than that in non-transformed HBE cells, and showed stronger nuclear localization characteristics (Supplementary Fig. S1a). We next explored the novel function of the abnormal expression of AURKA in tumor nuclei. Functional cluster analysis of the previously published AURKA-interacting proteins obtained by SILAC method^15^ showed that RNA alternative splicing regulation ranked first (Supplementary Fig. S1b–c, Supplementary Table 1), suggesting that nuclear AURKA may promote tumor progression by regulating RNA splicing.

In order to test this conjecture, we performed RNA-seq to identify the mRNA splicing events affected by AURKA knockdown in NCI-H460 cells. A total of 490 events were influenced by AURKA depletion (The intersection of two shAURKA groups; *P*-value < 0.05), including skipped exon (SE), alternative 3’ ss exon (A3E), alternative 5’ ss exon (A5E), mutually exclusive exons (MXE), and retained intron (RI) (Supplementary Fig. S2a, Supplementary Table 2). Skipped exon, the most common type of alternative splicing, accounts for 76.3% of total events. UniProt Keywords analysis was conducted to evaluate the functional classification of AURKA-regulated splicing events of SE type, revealing a significant enrichment of terms related to alternative splicing (Fig. 1b, Supplementary Table 3). To further identify targets specifically regulated by AURKA with RNA splicing regulatory functions, we overlapped AURKA-regulated SE events with AURKA-RIP-seq results and then compared these targets with RNA splicing-related genes^18^ (AURKA-RIP-seq database, Supplementary Table 4). Two RNA splicing-related candidates, SRRM1 and RBM4, were identified to be modulated by AURKA downregulation (Supplementary Fig. 1c). RBM4 is a splicing inhibitor that functions as a tumor suppressor through controlling cancer-related splicing,^19^ however, whether RBM4 itself is regulated by alternative splicing remains unknown. Surprisingly, here we found that RBM4 pre-mRNA could be spliced into a short isoform (RBM4-S), which contains only an N-terminal RNA-binding domain compared to the full-length isoform (RBM4-FL) (Fig. 1d). To determine the role of AURKA in the regulation of RBM4 splicing in lung cancer cells, we utilized Doxycycline (DOX)-induced Tet-On-shAURKA to deplete the expression of endogenous AURKA. Both RT-PCR and RT-qPCR analysis revealed that RBM4-S was substantially decreased and RBM4-FL was obviously increased following AURKA depletion in NCI-H460 and A549 cells (Fig. 1e–f, Supplementary Fig. S2b–c). Consistently, western blot analysis also corroborated that AURKA downregulation promoted RBM4 exon inclusion with or without RBM4 minigene reporter in NCI-H460 cells (Fig. 1g, Supplementary Fig. S2d).

We further investigated whether nuclear AURKA is required for RBM4 splicing regulation. To this end, we reconstituted AURKA-WT (wild-type), AURKA-NLS (nuclear-localized sequence), and AURKA-NES (nuclear export sequence) in endogenous AURKA depleted NCI-H460 and A549 cells. We found that nuclear relocalized AURKA could effectively inhibit AURKA knockdown-mediated RBM4 exon inclusion, whereas cytoplasmic accumulation of AURKA could not affect it, suggesting that nuclear translocation of AURKA plays an essential role in regulating RBM4 splicing switch (Fig. 1h–i, Supplementary Fig. S2e–f). In addition, inhibition of AURKA kinase activity with two different AURKA kinase inhibitors had no effect on RBM4 splicing switch, indicating AURKA modulates RBM4 splicing independent of its kinase activity (Supplementary Fig. S2g). Moreover, we conducted an RNA immunoprecipitation (RIP) assay to verify whether AURKA can bind to RBM4 pre-mRNA, and discovered that AURKA protein was able to bind both the exon and intron sequences of RBM4 pre-mRNAs in NCI-H460 and A549 cells (Fig. 1j, Supplementary Fig. S2h). Consistently, RNA fluorescent in situ hybridization (FISH) results revealed that AURKA protein co-localized with RBM4 pre-mRNAs in the nucleus (Fig. 1k). Taken together, these data indicate that nuclear AURKA drives RBM4 RNA splicing switch from RBM4-FL to RBM4-S by a kinase-independent mechanism in lung cancer.

### RBM4-S functions as a tumor promoter by abolishing RBM4-FL-mediated inhibition of SRSF1-mTORC1 activity

**Fig. 2:**
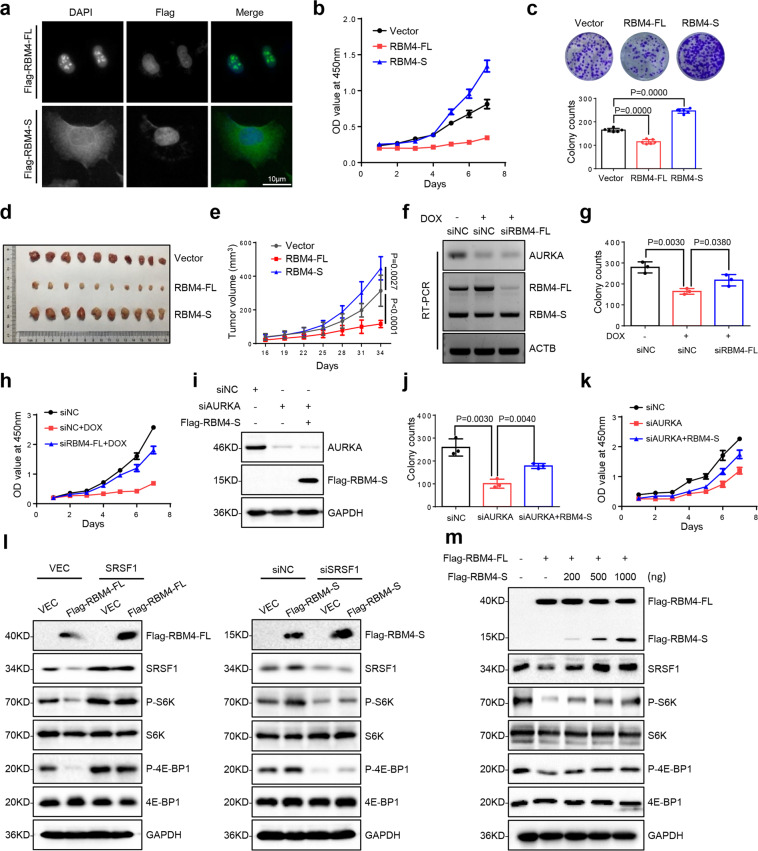
RBM4-S functions as a tumor promoter by abolishing RBM4-FL-mediated inhibition of SRSF1-mTORC1 activity. **a** Flag-labeled RBM4-FL and RBM4-S were transfected into A549 cells, respectively. The localization of Flag-RBM4-FL and Flag-RBM4-S was detected by IF assay with anti-Flag antibody (green). The nuclei were stained with DAPI (blue). Scale bar, 10 μm. The effects of RBM4-FL and RBM4-S on the proliferation of A549 cells. Cells were stably transfected with Vector, RBM4-FL, RBM4-S plasmids, and the proliferative ability of cells was analyzed by **b** cell counting kit-8 (CCK8) and **c** colony formation assays. **d**, **e** Immunodeficient mice were subcutaneously inoculated with equal number of Vector, RBM4-FL, and RBM4-S stably expressing A549 cells (2 × 10^6^ cells per mouse, *n* = 10). Photograph and tumor volume were shown. **f** Validation of the expression of AURKA and two variants of RBM4 by RT-PCR. **g**, **h** Colony formation and CCK8 proliferative assays were conducted to examine cell proliferation ability. **i** Validation of the expression of AURKA and exogenous Flag-RBM4-S by western blot. **j**, **k** Colony formation and CCK8 proliferative assays were performed to examine cell proliferation ability. **l** SRSF1 was transiently expressed in control and Flag-RBM4-FL overexpressed A549 cells, and SRSF1 was transiently depleted in control and Flag-RBM4-S overexpressed A549 cells. The protein expression levels of Flag-RBM4-FL or Flag-RBM4-S, SRSF1, P-4E-BP1, 4E-BP1, P-S6K, and S6K were assessed by western blot assay and normalized to GAPDH. **m** Increasing amounts of Flag-RBM4-S were co-expressed with Flag-RBM4-FL in HEK-293T cells. Flag-RBM4-FL/S, SRSF1, P-4E-BP1, 4E-BP1, P-S6K, and S6K protein expression levels were assessed by western blot and normalized to GAPDH. Data are shown as means ± SD. *P*-values were calculated with two-tailed unpaired Student’s *t*-test and *P* < 0.05 is considered statistically significant

Since the mouse *RBM4* gene has 95% homology with the human orthologue,^20^ we firstly explored whether RBM4-S isoform is widely expressed in mouse tissues. RT-PCR results showed that RBM4-S expresses in a tissue-specific manner (Supplementary Fig. S3a). To further investigate the function of the newly discovered RBM4-S isoform, we exogenously overexpressed Flag tag labeled RBM4-S and RBM4-FL plasmids into A549 cells (Supplementary Fig. S3b). Intriguingly, the two RBM4 isoforms have distinct subcellular localizations: RBM4-FL is primarily located in the nuclear speckles, whereas RBM4-S is preferentially expressed in the cytoplasm (Fig. 2a). Similar results were observed in H1299 cells (Supplementary Fig. S3c). Subsequently, we examined the effects of these two RBM4 isoforms on the proliferation of a variety of human cancer cells, including A549 and NCI-H460 (lung cancer), MDA-MB-231 (breast cancer), and HepG2 (liver cancer). Colony formation and CCK8 assays evidently demonstrated that overexpressing RBM4-FL suppressed, whereas RBM4-S promoted, cell proliferation in all cancer cells tested (Fig. 2b–c, Supplementary Fig. S4a–c). Consistently, we further verified the opposite functions of the two RBM4 isoforms on tumor growth in vivo. Mice inoculated with A549-RBM4-FL cells showed a significant reduction in the tumor growth as indicated by the decreased tumor volume when compared with mice bearing A549-Vector control cells. In contrast, tumors derived from A549-RBM4-S cells grew at a significantly increased rate compared to those from A549-Vector cells (Fig. 2d–e). In addition, both depletion of RBM4-FL and overexpression of RBM4-S in AURKA knockdown cells could at least partially reverse the decreased cell proliferative activity caused by AURKA suppression (Fig. 2f–k, Supplementary Fig. S4d–e). Altogether, these results demonstrate that RBM4-S plays an opposite role as compared to the canonical tumor suppressor RBM4-FL, therefore, the splicing switch towards RBM4-S is partially responsible for AURKA-regulated tumor growth.

We next investigated the underlying molecular mechanisms of how RBM4-S promotes cancer cell proliferation. As RBM4-S preserved N-terminal RNA-binding domain compared with RBM4-FL, we first examined if RBM4-S could interfere with the classical splicing regulatory function of RBM4-FL. We co-expressed RBM4-FL and increased amounts of RBM4-S with splicing reporters containing RBM4 binding site,^19^ and found that RBM4-S expression had no effect on the splicing inhibitory activity of RBM4-FL (Supplementary Fig. S5a–b). Our previous studies have demonstrated that the anticancer activity of RBM4 depends not only on its splicing regulatory function but also on its antagonized effect on the expression of oncogenic SRSF1 protein.^19^ SRSF1 plays a critical oncogenic role by activating the mTORC1 pathway in both alternative splicing-dependent and -independent manner, as measured by S6K1 and 4E-BP1 phosphorylation.^21–24^ Therefore, we sought to determine if RBM4-S could influence SRSF1 protein expression. Intriguingly, overexpressing RBM4-S had an opposite effect on SRSF1 protein expression as compared to RBM4-FL overexpressed cells (Supplementary Fig. S5c). Moreover, both RBM4-FL overexpression mediated mTORC1 signaling inhibition and RBM4-S overexpression mediated mTORC1 signaling activation was dependent on the protein level of SRSF1 (Fig. 2l). Co-expression of RBM4-FL with increased amounts of RBM4-S substantially reversed RBM4-FL-induced downregulation of the oncogenic SRSF1 protein, further activating SRSF1-mediated mTORC1 signaling pathway in a dose-dependent manner, as judged by the significantly increased phosphorylation levels of S6K1 and 4E-BP1 (Fig. 2m). In addition, overexpression of RBM4-S in DOX-induced AURKA knockdown cells reverted the AURKA depletion-induced downregulation of SRSF1 (Supplementary Fig. S5d). Collectively, these results indicate that RBM4-S functions as a tumor promoter by abolishing RBM4-FL-mediated inhibition of SRSF1-mTORC1 signaling pathway activity.

### Nuclear AURKA inhibits m^6^A-YTHDC1-SRSF3-mediated RBM4 exon inclusion

**Fig. 3:**
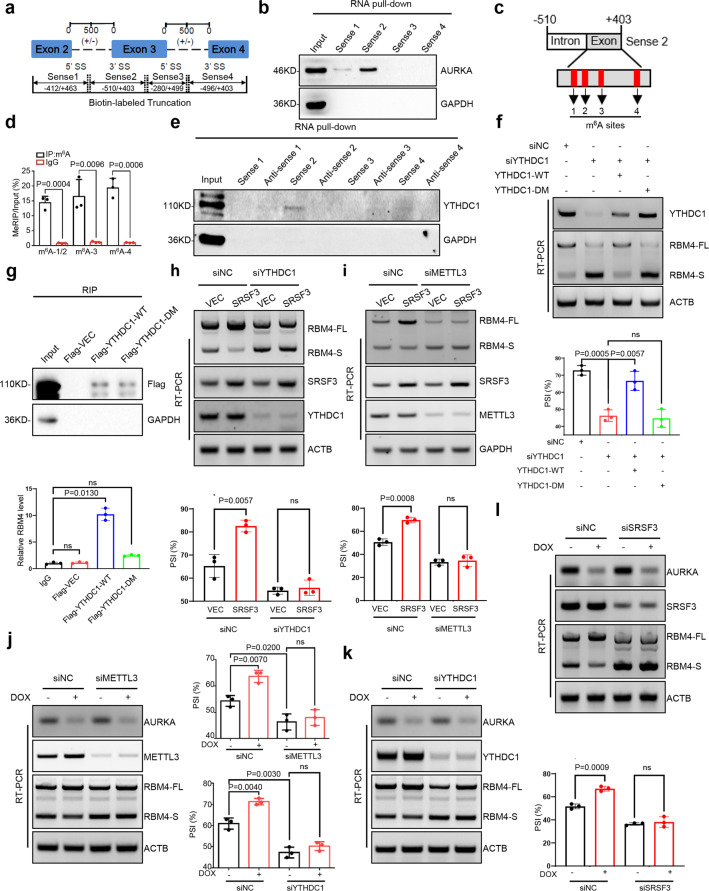
Nuclear AURKA inhibits m^6^A-YTHDC1-SRSF3-mediated RBM4 exon inclusion. **a** *RBM4* gene was truncated into four parts, each of which was applied to an in vitro transcription assay with Biotin-labeled as indicated. Sense 1: −412/+463, Sense 2: −510/+403, Sense 3: −280/+499, Sense 4: −496/+403. **b** The streptavidin beads-immobilized biotin-labeled RNA truncations were incubated with A549 cell lysates and an RNA pull-down assay was performed. **c** Four potential m^6^A sites located in the Sense 2 (−510/+403) region of RBM4 pre-mRNA. **d** RNA methylation modification was detected by the methylated RNA-immunoprecipitation (MeRIP)-qPCR assay in A549 cells. **e** The PCR products of RBM4 truncated sense and anti-sense regions were applied to an in vitro transcription assay with Biotin-labeled. Binding of these RNA truncations with YTHDC1/GAPDH proteins was examined by an RNA pull-down assay in A549 cells. **f** RBM4 splicing change in YTHDC1-WT (wild-type) and YTHDC1-DM (m^6^A binding region mutant) reconstitution endogenous YTHDC1 depleted A549 cells was detected by RT-PCR. **g** Binding of RBM4 pre-mRNA with IgG or exogenous Flag-VEC/Flag-YTHDC1-WT/Flag-YTHDC1-DM protein was detected by RIP-qPCR assay in A549 cells. **h** The level of YTHDC1 or **i** METTL3 was depleted in control and SRSF3 overexpressed A549 cells. Relative mRNA abundance of SRSF3, YTHDC1/METTL3, and RBM4 splicing was examined by RT-PCR. **j** The level of METTL3 or **k** YTHDC1 or **l** SRSF3 was depleted in A549-Tet-On-shAURKA cells with or without DOX induction. Relative mRNA abundance of AURKA, METTL3/YTHDC1/SRSF3, and RBM4 splicing was examined by RT-PCR. Data are shown as means ± SD. *P*-values were calculated with two-tailed unpaired Student’s *t*-test and *P* < 0.05 is considered statistically significant

To explore the mechanism of AURKA regulating RBM4 aberrant RNA splicing, we first studied the potential AURKA binding region on RBM4 pre-mRNA. *RBM4* gene was truncated into four parts, each of which was applied to an in vitro transcription assay with Biotin-labeled at N-terminus (Fig. 3a). RNA pull-down results demonstrated that the region of sense 2 (−510/+403) was bound by AURKA protein (Fig. 3b). To further analyze the characteristics of the sense 2 region, we found that RNA methylation sites were highly accumulated in this region by using SRAMP (a computational predictor of mammalian m^6^A site) (Supplementary Fig. S6a). Consistently, four potential RNA methylation sites were predicted by RMBase v2.0 database^25^ (Fig. 3c). MeRIP-qPCR assay proved that all the potential sites could be modified by methylation (Fig. 3d). We next investigated whether m^6^A and its readers were involved in RBM4 splicing regulation. Among a variety of m^6^A readers, YTHDC1, hnRNP A2B1, hnRNP C, and IGF2BP3 have been reported to regulate RNA splicing.^13,26–28^ In order to investigate which m^6^A reader regulates the alternative splicing of RBM4, we conducted RT-PCR screening and found that only YTHDC1 knockdown can inhibit RBM4 exon inclusion (Supplementary Fig. S6b). Moreover, RNA pull-down assay revealed that the truncated sense 2 region of RBM4 pre-mRNA was associated with m^6^A reader YTHDC1 (Fig. 3e). RT-qPCR data showed that depletion of YTHDC1 in A549 cells promoted RBM4 exon skipping, which was similar to the effect of METTL3 knockdown (Supplementary Fig. S6c–d). Western blot analysis also showed that both YTHDC1 and METTL3 reduction in A549 cells switched the splicing of RBM4 minigene reporter towards the RBM4-S isoform (Supplementary Fig. S6e–f). To further validate that YTHDC1 regulates RBM4 splicing via recognizing m^6^A, on the one hand, we reconstituted YTHDC1-WT (wild-type) and YTHDC1-DM (m^6^A binding region mutant) in YTHDC1 depleted A549 cells, and found that re-expression of YTHDC1 wild-type, but not mutant, could rescue YTHDC1 knockdown-mediated RBM4 exon 3 skipping (Fig. 3f). On the other hand, RIP data showed that the wild-type of YTHDC1, but not the mutant, can bind to RBM4 mRNA (Fig. 3g). These data suggest that YTHDC1 promotes RBM4 exon 3 inclusion in an m^6^A-dependent manner.

However, recent studies have shown that YTHDC1 plays a regulatory role in RNA alternative splicing via recruiting other splicing factors.^12,13^ Xiao et al. demonstrated that YTHDC1 recruitment of SRSF3 facilitates SRSF3 to perform RNA splicing function.^12,13^ To verify this, we first knocked down and overexpressed SRSF3 in lung cancer cells and found that SRSF3 promoted RBM4 exon 3 inclusion (Supplementary Fig. S7a). RIP data also showed that SRSF3 protein was able to bind RBM4 pre-mRNA (Supplementary Fig. S7b). We then found that depletion of YTHDC1 in SRSF3 overexpressed A549 cells could significantly reverse SRSF3 upregulation-mediated RBM4 exon inclusion (Fig. 3h, Supplementary Fig. S7c). Similarly, suppressing METTL3 expression in SRSF3 overexpressed A549 cells abolished SRSF3 upregulation-induced RBM4-FL production (Fig. 3i, Supplementary Fig. S7d), suggesting that SRSF3 promotes RBM4 exon inclusion dependent on m^6^A reader YTHDC1. These data indicate that RBM4 splicing towards RBM4-FL depends on the m^6^A site’s recognition by the YTHDC1-SRSF3 complex.

To confirm whether the m^6^A-YTHDC1-SRSF3 complex is involved in AURKA-regulated RBM4 splicing, we conducted siRNA-mediated METTL3, YTHDC1 and SRSF3 depletion in DOX-induced AURKA knockdown A549 cells. Importantly, downregulation of METTL3 or YTHDC1 or SRSF3 abolished AURKA depletion-induced splicing switch towards RBM4-FL in A549 cells by RT-PCR and RT-qPCR analysis (Fig. 3j–l, Supplementary Fig. S8a–c), suggesting that AURKA inhibits m^6^A-YTHDC1-SRSF3 complex-mediated RBM4 exon inclusion.

### Nuclear AURKA interacts with m^6^A reader YTHDC1 and disrupts the binding of SRSF3 to YTHDC1

**Fig. 4:**
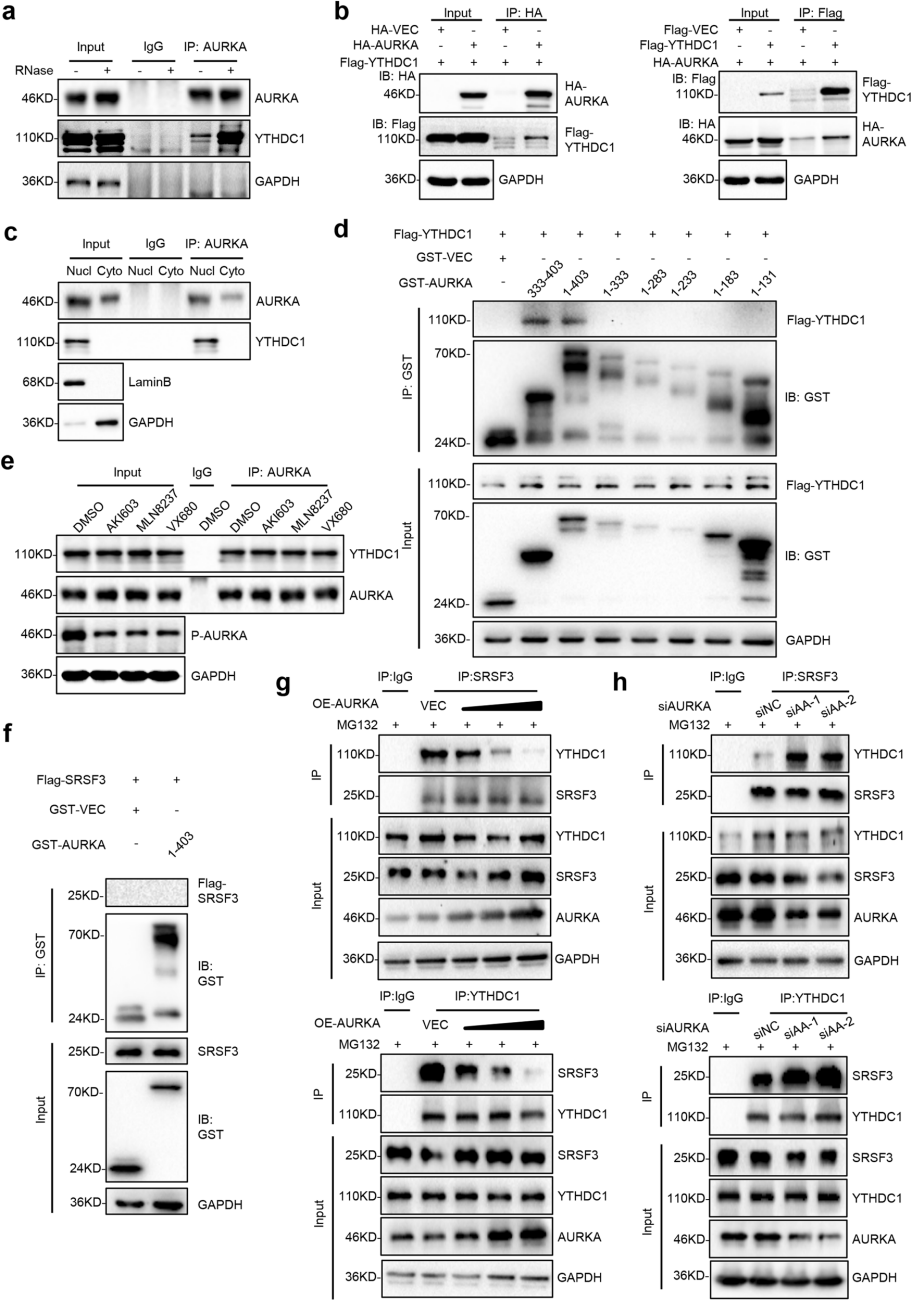
Nuclear AURKA interacts with m^6^A reader YTHDC1 and disrupts the binding of SRSF3 to YTHDC1. **a** The interaction between endogenous AURKA and YTHDC1 in both the absence and presence of RNase was analyzed by a co-immunoprecipitation (Co-IP) assay. **b** The interaction between exogenous HA-AURKA and Flag-YTHDC1 was measured by Co-IP assay in HEK-293T cells. **c** Nuclear/cytoplasmic protein fractions of A549 cells were subjected to immunoprecipitation (IP) and immunoblotting (IB) using antibodies as indicated. **d** GST-fused AURKA full-length (1–403 aa) and truncations (333–403 aa, 1–333 aa, 1–283 aa, 1–233 aa, 1–183 aa, 1–131 aa) were co-expressed with Flag-YTHDC1 in HEK-293T cells. Protein interaction was analyzed by the GST pull-down assay. **e** The protein interaction between AURKA and YTHDC1 was analyzed by a Co-IP assay in A549 cells treated with DMSO and three kinds of AURKA kinase inhibitors (AKI603/MLN8237/VX680). AKI603, 1 μM; MLN8237, 0.2 μM; VX680, 0.5 μM. **f** GST-fused AURKA full-length (1–403 aa) was co-expressed with Flag-SRSF3 in HEK-293T cells. Protein interaction was analyzed by the GST pull-down assay. **g** Co-IP analysis of the protein interaction between YTHDC1 and SRSF3 in NCI-H460 cells transfected with increasing concentrations of plasmid encoding AURKA after MG132 treatment. MG132, 20 mM. **h** Co-IP analysis of the protein interaction between SRSF3 and YTHDC1 in NCI-H460 cells transfected with different siRNAs to knockdown AURKA after MG132 treatment. MG132, 20 mM

To further mechanistically study the AURKA-modulated YTHDC1-SRSF3 complex-dependent RBM4 splicing, we sought to investigate the relationship between AURKA and YTHDC1-SRSF3 complex. Nuclear extracts from A549 cells were treated with RNase A to remove RNAs, thereby disrupting RNA-mediated interactions. The subsequent Co-IP assay data showed that YTHDC1 could be pulled down by AURKA in both the absence and presence of RNase A (Fig. 4a), implying that the AURKA-YTHDC1 interaction primarily involves protein-protein interaction. Consistently, the exogenously expressed HA-tagged AURKA was able to be co-precipitated with Flag-tagged YTHDC1, and vice versa (Fig. 4b). In addition, Co-IP assays performed with nuclear extraction demonstrated that the interaction between AURKA and YTHDC1 was predominantly located in the nucleus (Fig. 4c). To identify the essential domain of AURKA for interacting with YTHDC1, full-length (1–403 aa) and truncated isoforms (333–403 aa, 1–333 aa, 1–283 aa, 1–233 aa, 1–183 aa, 1–131 aa) of AURKA were fused to glutathione S-transferase (GST). The following GST pull-down assay showed that only AURKA full-length (1–403 aa) and 333–403 truncated isoform could bind to YTHDC1, whereas other regions (1–333 aa, 1–283 aa, 1–233 aa, 1–183 aa, 1–131 aa) were not capable of binding to YTHDC1, suggesting that YTHDC1 interacts with AURKA 333–403 region (Fig. 4d). Targeting AURKA kinase activity with three different AURKA kinase inhibitors failed to disrupt the binding of AURKA to YTHDC1, implying such interaction is not in a kinase activity-dependent manner (Fig. 4e). In addition, GST pull-down assay revealed that AURKA could not bind to SRSF3 directly (Fig. 4f). Critically, we further found that overexpression of AURKA could inhibit the protein interaction between SRSF3 and YTHDC1 in a dose-dependent manner (Fig. 4g). Inhibition of AURKA expression obviously promoted the protein binding of SRSF3 to YTHDC1 (Fig. 4h). Taken together, these data indicate that nuclear AURKA antagonizes the binding of SRSF3 to YTHDC1.

### Nuclear AURKA bridges the interaction between hnRNP K and YTHDC1 and promotes m^6^A-YTHDC1-hnRNP K-dependent RBM4 exon skipping

**Fig. 5:**
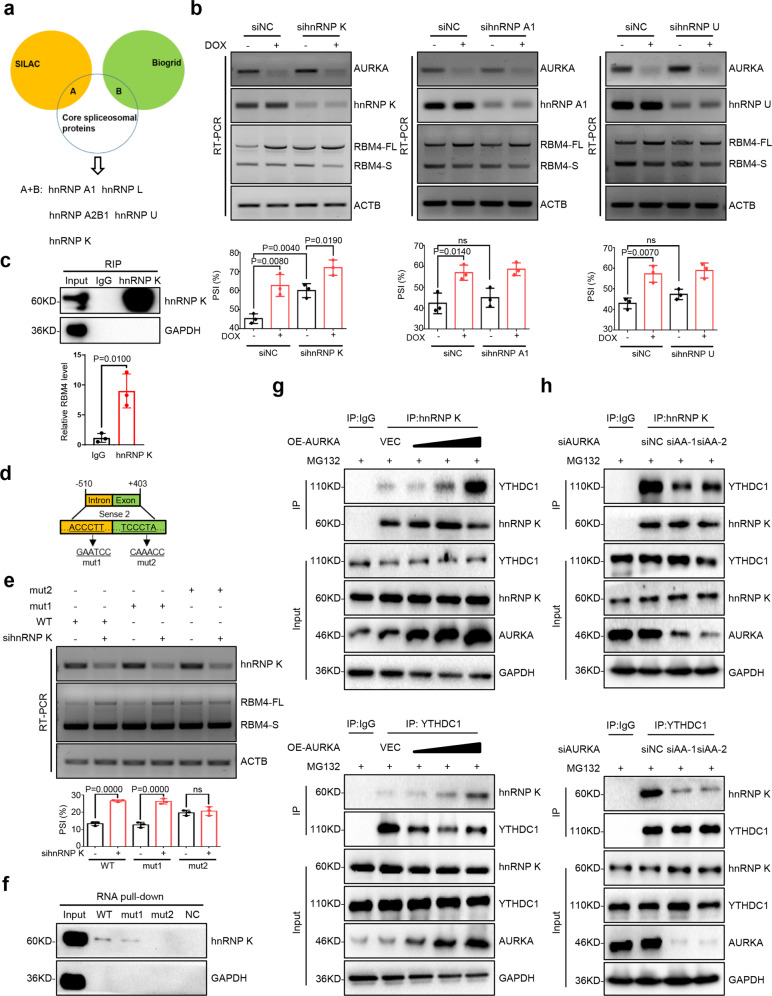
Nuclear AURKA bridges the interaction between hnRNP K and YTHDC1 and promotes m^6^A-YTHDC1-hnRNP K-dependent RBM4 exon skipping. **a** Combination of AURKA-interacting proteins from SILAC and Biogrid database were compared with core spliceosomal proteins. **b** The level of hnRNP K, hnRNP A1 and hnRNP U was depleted respectively in A549-Tet-On-shAURKA cells with or without DOX induction. Relative mRNA abundance of AURKA, hnRNPs, and RBM4 splicing was examined by RT-PCR. **c** Binding of RBM4 pre-mRNA with IgG or hnRNP K protein was detected by RIP-qPCR assay in A549 cells. **d** Two potential hnRNP K binding sites located in the Sense 2 (−510/+403) region of RBM4 pre-mRNA. RBM4 splicing reporters with the indicated mutations (mut1 and mut2) were generated. **e** RBM4 splicing reporters containing wild-type (WT) and mutations (mut1 or mut2) were overexpressed in A549 cells with siNC or sihnRNP K. RT-PCR was conducted to detect the splicing change of RBM4. **f** The PCR products of negative control GAPDH (NC) and hnRNP K binding sites (WT/mut1/mut2) were applied to an in vitro transcription assay with Biotin-labeled. Binding of these RNAs with hnRNP K/GAPDH proteins was detected by an RNA pull-down assay in A549 cells. **g** Co-IP analysis of the protein interaction between YTHDC1 and hnRNP K in NCI-H460 cells transfected with increasing concentrations of plasmid encoding AURKA after MG132 treatment. MG132, 20 mM. **h** Co-IP analysis of the protein interaction between hnRNP K and YTHDC1 in NCI-H460 cells transfected with different siRNAs to knockdown AURKA after MG132 treatment. MG132, 20 mM. Data are shown as means ± SD. *P*-values were calculated with two-tailed unpaired Student’s *t*-test and *P* < 0.05 is considered statistically significant

To further explore the mechanism of AURKA-mediated RBM4 splicing towards RBM4-S, we overlapped core spliceosomal proteins^29^ with both the AURKA SILAC database and AURKA-interacting proteins published in Biogrid.^30^ We then combined two groups of overlapping proteins together, and found that AURKA could be potentially bound by multiple splicing factors, including hnRNP A1, hnRNP L, hnRNP A2B1, hnRNP U, and hnRNP K (Fig. 5a). To identify the hnRNPs involved in AURKA-regulated RBM4 splicing, individual hnRNP members were transiently knocked down in A549-Tet-On-shAURKA cells with or without DOX induction. Notably, only depletion of hnRNP K significantly inhibited RBM4 exon 3 skipping (Fig. 5b, Supplementary Fig. S9a). When knocking down both AURKA and hnRNP K, the PSI value of RBM4 was much higher than that in single silencing cells (Fig. 5b). RIP assay also showed that hnRNP K protein could interact with RBM4 pre-mRNA (Fig. 5c). To confirm the scissor effect of splicing factor hnRNP K, we next sought to verify two potential hnRNP K binding sites predicted by SpliceAid,^31^ which located at the sense 2 (−510/+403) region of RBM4 pre-mRNA (Fig. 5d). Using RBM4 minigene reporters containing distinct mutations, we found that hnRNP K-mediated RBM4 splicing switch was dependent on the second predicted splice site (TCCCTA), as mutation of this site fully abolished RBM4 exon inclusion upon hnRNP K depletion (Fig. 5e). Consistently, RNA pull-down data demonstrated that the motif TCCCTA was responsible for the interaction between hnRNP K and RBM4 pre-mRNA (Fig. 5f). These data demonstrate that hnRNP K, acting as another pair of scissors, directly promotes RBM4 exon skipping via recognizing the splice site (TCCCTA) of RBM4 pre-mRNA.

To further mechanistically study how AURKA-hnRNP K complex works, we need to explore the relationship between hnRNP K, AURKA and YTHDC1. We found that hnRNP K not only can interact with AURKA,^15^ but also can co-precipitate with YTHDC1 (Fig. 5g–h). Importantly, overexpression of AURKA strikingly enhanced the protein interaction between hnRNP K and YTHDC1 in a dose-dependent manner (Fig. 5g). While silencing AURKA expression inhibited the protein binding of hnRNP K to YTHDC1 (Fig. 5h). Collectively, these data suggest that nuclear AURKA recruits the splicing factor hnRNP K to YTHDC1 and promotes m^6^A-YTHDC1-hnRNP K-dependent RBM4 exon skipping.

### AURKA nuclear translocation inhibitors reverse RBM4 splicing towards RBM4-S to restrain tumor growth

**Fig. 6:**
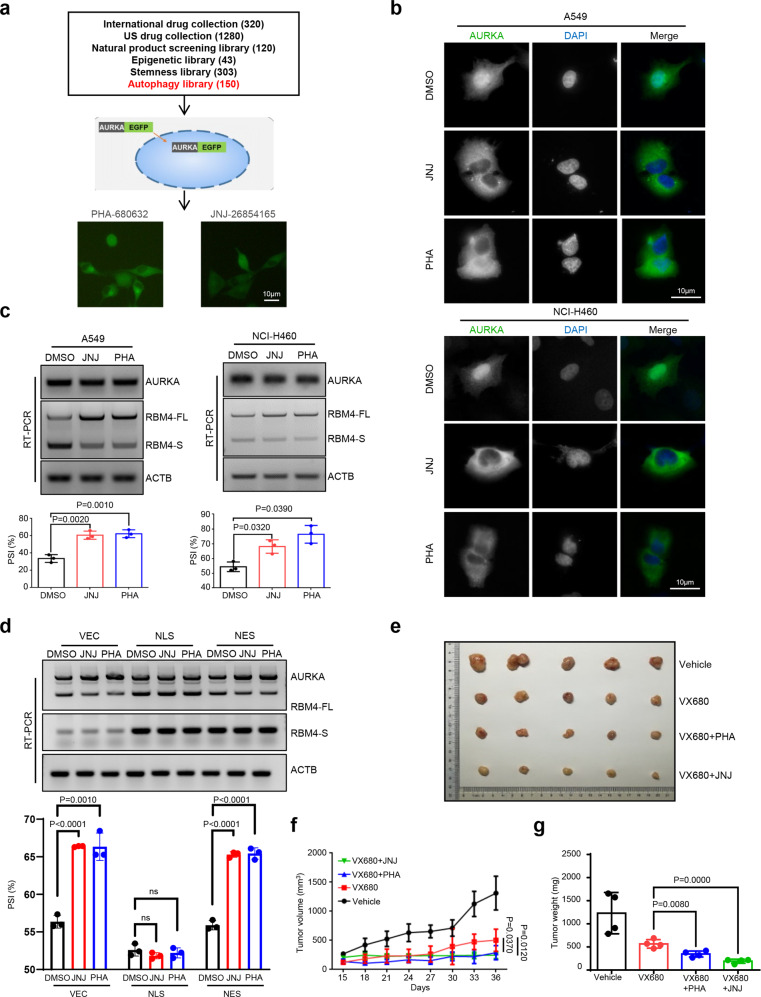
AURKA nuclear translocation inhibitors reverse RBM4 splicing towards RBM4-S to restrain tumor growth. **a** Establishment of small-molecule chemical libraries and drug screening model. The localization of enhanced green fluorescent protein (EGFP)-fused AURKA was analyzed via inverted fluorescence microscope. Scale bar, 10 μm. PHA, 10 μM; JNJ, 10 μM. **b** The localization of endogenous AURKA protein (green) was detected by IF assay in A549 and NCI-H460 cells treated with DMSO, JNJ, and PHA, respectively. The nuclei were stained with DAPI (blue). Scale bar, 10 μm. **c** The splicing change of RBM4 was validated by RT-PCR in A549 and NCI-H460 cells treated with DMSO, JNJ and PHA. **d** Empty vector, AURKA-NLS, and AURKA-NES were overexpressed in NCI-H460 cells treated with DMSO, JNJ, and PHA. RT-PCR was conducted to assay for the splicing change of RBM4. **e**–**g** Immunodeficient mice were subcutaneously inoculated with equal number of NCI-H460 cells (6 × 10^6^ cells per mouse). When tumor size reached 100 mm^3^, animals were randomized into four groups (*n* = 5/group) and treated as follows: PBS, VX680 (20 mg/kg/d), PHA (40 mg/kg/d) combined with VX680 (20 mg/kg/d), JNJ (20 mg/kg/d) combined with VX680 (20 mg/kg/d). The agents were administered by gavage and the treatment continued for 10 days. Photograph of tumors was shown. Tumor weight and volume were shown. Data are shown as means ± SD. *P-*values were calculated with two-tailed unpaired Student’s *t*-test and *P* < 0.05 is considered statistically significant

Since nuclear AURKA plays a vital role in cancer development, we screened six drug libraries containing International drug collection (320), US drug collection (1280), Natural product screening library (120), Epigenetic library (43), Stemness library (303), and Autophagy library (150) in the EGFP-AURKA stable expression system to identify small molecules that could efficiently block AURKA nuclear translocation (Fig. 6a). Intriguingly, we discovered two chemicals that were capable of preventing AURKA from translocating into the nucleus, including JNJ-26854165 (JNJ), an E3 ligase inhibitor, and PHA-680632 (PHA), an Aurora kinase inhibitor, both of which came from the Autophagy library (Fig. 6a). To further verify the effect of these two chemicals on endogenous AURKA nuclear translocation, we performed IF assay and found that both JNJ and PHA facilitated cytoplasm accumulation of AURKA in A549 and NCI-H460 cells (Fig. 6b).

Subsequently, we investigated whether JNJ and PHA could specifically modulate RBM4 splicing. We examined the expression level and kinase activity of AURKA in response to these two chemicals in A549 and NCI-H460 cells. Our results showed that JNJ and PHA inhibited AURKA kinase activity, but had no effect on the total expression level of AURKA (Supplementary Fig. S10a). We have confirmed that AURKA kinase inhibitors had no effect on RBM4 splicing in lung cancer cells (Supplementary Fig. S2g). Further data revealed that both JNJ and PHA treatment enhanced the exon 3 inclusion of RBM4 in A549 and NCI-H460 cells (Fig. 6c). To validate whether nuclear AURKA plays a key role in JNJ and PHA mediated RBM4 splicing switch, we overexpressed AURKA-NLS and AURKA-NES in control and drug treatment groups, respectively. The results demonstrated that nuclear accumulation of AURKA, but not cytoplasmic accumulation, abolished JNJ and PHA treatment-induced RBM4 exon inclusion (Fig. 6d). These data indicate that JNJ and PHA promote RBM4 exon inclusion through blocking AURKA nuclear translocation.

To determine the in vivo effects of JNJ and PHA on tumor growth, an equal number of NCI-H460 cells (6 × 10^6^) were subcutaneously injected into 4–6-weeks-old BALB/C (nu/nu) female nude mice, and the mice were randomly divided into four groups after the tumor volume reached more than 100 mm^3^. DMSO, VX680, VX680 + PHA, VX680 + JNJ were subsequently administered by gavage once a day for 10 days. Remarkably, tumor growth, as indicated by tumor volume and weight, was significantly suppressed with the combined drugs as compared to VX680 alone and the control group (Fig. 6e–g). Moreover, the production of RBM4-FL isoform was elevated in the combined drugs groups than that in VX680 alone and the control group (Supplementary Fig. S10b). Together, these data demonstrate that small-molecule AURKA nuclear translocation inhibitors JNJ and PHA suppress RBM4 splicing towards RBM4-S, thereby restraining tumor growth.

### Clinical relevance of AURKA-RBM4 aberrant splicing axis

**Fig. 7:**
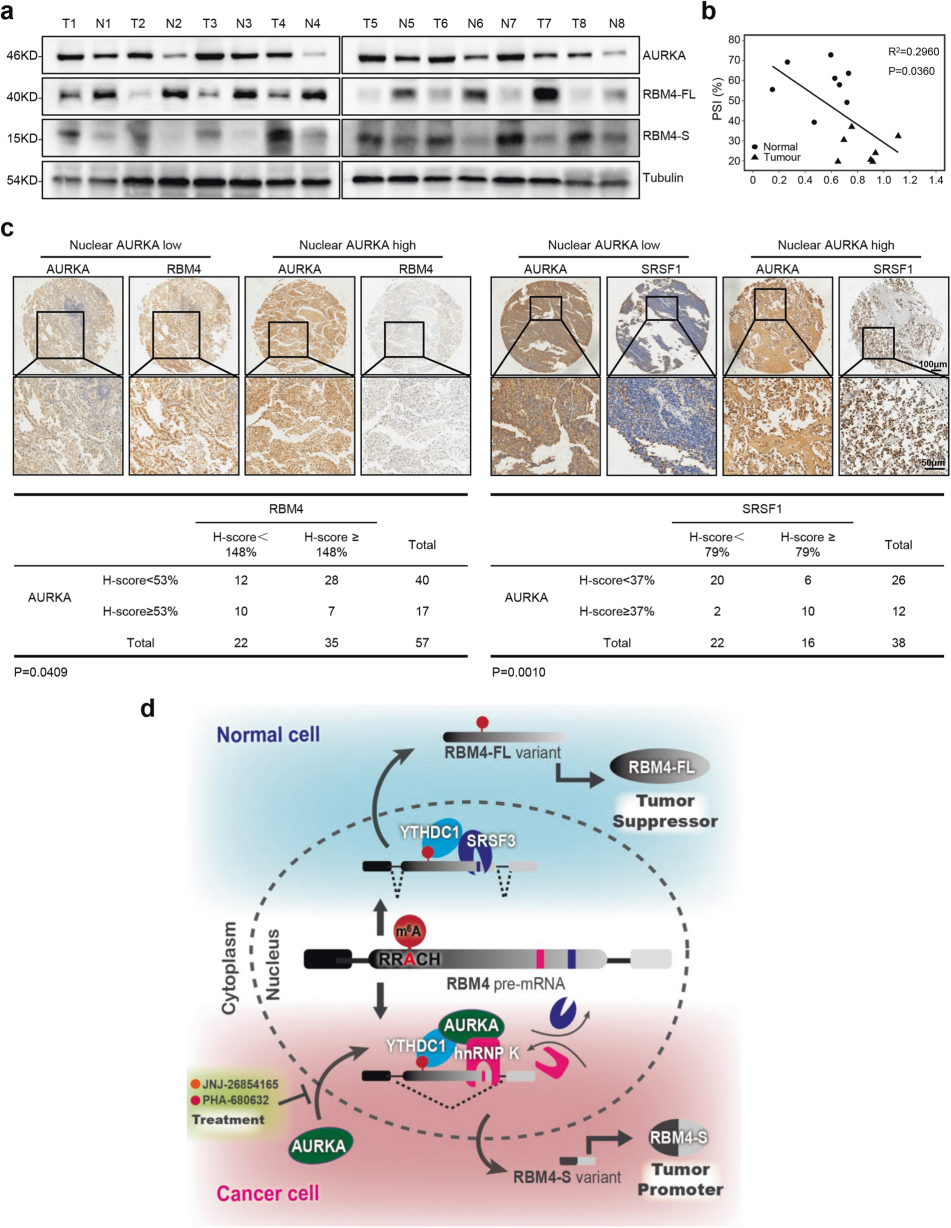
Clinical relevance of AURKA-RBM4 aberrant splicing axis. **a** Relative protein expression levels of AURKA and RBM4-FL/S in eight pairs of NSCLC specimens (T) and adjacent normal tissues (N) were subjected to western blot analysis. **b** Linear regression analysis of a correlation between AURKA relative expression level and the PSI value of RBM4 in eight pairs of clinical specimens was shown. R^2^ = 0.2960, *P* = 0.0360. **c** Representative images of nuclear AURKA, RBM4, and SRSF1 IHC staining in lung tumor specimens. Scale bars, 100 μm or 50 μm. **d** Schematic diagram of the mechanism: Nuclear AURKA disrupts the binding of SRSF3 to m^6^A reader YTHDC1, thereby inhibiting the production of m^6^A-YTHDC1-SRSF3-mediated tumor suppressive isoform RBM4-FL. In turn, nuclear AURKA bridges the interaction between hnRNP K and YTHDC1, thus facilitating the production of m^6^A-YTHDC1-hnRNP K-mediated tumor-promoting isoform RBM4-S. Blocking AURKA nuclear translocation, can reverse the oncogenic splicing of RBM4 and suppress lung tumor progression. Pearson’s correlation test in **c**. *P* < 0.05 is considered statistically significant

To explore the clinical relevance of the AURKA-RBM4 aberrant splicing axis, we collected 8 pairs of non-small cell lung cancer (NSCLC) specimens and adjacent normal tissues to measure the protein levels of AURKA and RBM4 two splicing isoforms. Compared with adjacent normal tissues, the relative protein levels of AURKA and RBM4-S were evidently increased in tumor tissues, while the relative protein levels of RBM4-FL were evidently decreased in tumor tissues (Fig. 7a). The subsequent linear regression analysis demonstrated a negative correlation between AURKA expression and the PSI value of RBM4 (R^2^ = 0.2960, *P* = 0.0360) in 8 pairs of clinical specimens (Fig. 7b). We then performed IHC staining of AURKA, RBM4 (RBM4-FL) and SRSF1 to determine their correlations in lung tumor tissues. Nuclear AURKA expression negatively correlated with the expression of RBM4-FL and positively correlated with the expression of SRSF1 (Fig. 7c). Hence, our data indicate that high expression of AURKA in the nucleus is positively associated with RBM4 aberrant splicing in lung cancer.

## Discussion

It is well-established that aberrant RNA splicing can produce abnormal proteins that contribute to cancer development. Till now, how this process is triggered by oncogenic signaling, however, has remained elusive. Here, we demonstrate a non-canonical function of nuclear AURKA to trigger an oncogenic RNA splicing of tumor suppressor RBM4 in a novel m^6^A reader YTHDC1-dependent mechanism in lung cancer cells, thereby providing a promising avenue for lung cancer therapeutics by targeting nuclear oncogenic signals. Nuclear translocation of AURKA acquires the kinase-independent function to promote RBM4 aberrant RNA splicing from the full isoform (RBM4-FL) to the short isoform (RBM4-S). RBM4-S enhances lung cancer cell proliferation by antagonizing RBM4-FL-meditated tumor suppression through eliminating the inhibition effect of RBM4-FL on the SRSF1-mTORC1 signaling pathway. Mechanistically, nuclear AURKA disrupts the binding of SRSF3 to YTHDC1 and bridges the interaction between hnRNP K and YTHDC1, resulting in the inhibition of m^6^A-YTHDC1-SRSF3-mediated RBM4 exon inclusion and the promotion of m^6^A-YTHDC1-hnRNP K-dependent RBM4 exon skipping. Furthermore, we show that AURKA nuclear translocation inhibitors could significantly suppress lung tumor progression by reversing the oncogenic splicing of RBM4.

Being an evolutionally highly conserved serine/threonine kinase essential for mitosis, AURKA plays a critical oncogenic role in promoting tumor initiation and development.^14^ Till now, a lot of efforts have been made to develop inhibitors against its kinase activity. However, the efficacy of AURKA kinase inhibitors such as Alisertib has been still very poor in clinical trials.^32,33^ The oncogenic function of AURKA is not completely blocked by the inhibition of its kinase activity, suggesting that AURKA may have non-classical kinase-independent carcinogenicity. Recent studies have shown that AURKA can function biologically in a kinase-independent manner. For example, AURKA promotes replication assembly during DNA replication initiation by kinase-independent mechanism and participates in the regulation of G1-S cell cycle.^34^ Defects in spindle assembly during mitosis induced by AURKA deletion can be rescued by the kinase-dead mutant.^35^ AURKA binds to N-MYC^36,37^ and C-MYC^38^ proteins in the MYC family, protecting them from proteasome pathway degradation. Our previous studies have shown that AURKA enhances breast cancer stem-like properties via its kinase-independent mechanism by (1) promoting the transcription of MYC and FOXM1 downstream target genes, (2) stabilizing DROSHA mRNA level,^15–17^ suggesting that the non-kinase-dependent function of AURKA is essential for the development of tumors. In this project, two observations suggest that AURKA may promote tumor progression by regulating RNA alternative splicing. One is that AURKA expression is highly associated with spliceosome activity by Pearson correlation analysis of whole-genome gene expression profile (clinical breast cancer samples from TCGA and METABRIC) with spliceosome activity score (Supplementary Fig. S11a–b, Supplementary Table 5). The other one is that RNA splicing regulation ranks first by the function clustering analysis of AURKA-interacting protein (Supplementary Fig. S1b–c). RNA-seq screening and genetic studies reveal that AURKA, frequently overexpressed and accumulated in nuclei during lung carcinogenesis, triggers RBM4 aberrant splicing from the full isoform (RBM4-FL) to the short isoform (RBM4-S), unexpectedly, in a kinase-independent way. These findings suggest that exploring the non-canonical activity of AURKA, namely studying the action and mechanism of abnormal RNA splicing mediated by AURKA, will provide new opportunities for targeted therapy of lung cancer.

N^6^-methyladenosine (m^6^A) represents one of the most prevalent internal RNA modifications in eukaryotes.^39^ m^6^A is recognized by its readers, such as YTH domain-containing proteins, to regulate mRNA metabolic processes, including RNA splicing.^13,40–44^ YTHDC1, as a critical nuclear m^6^A reader, localizes in YT-bodies adjacent to nuclear speckles. *YTHDC1*-deficient oocytes exhibit widespread splicing defects, which could be mostly rescued by supplementing with wild-type YTHDC1, but not m^6^A binding domain mutant.^13^ In our project, we find that RBM4 pre-mRNA can be highly methylated and bind to YTHDC1 protein (Fig. 3c–e). Reconstruction of YTHDC1 wild-type, but not m^6^A binding site mutant, could rescue YTHDC1 knockdown-mediated RBM4 exon skipping (Fig. 3f). Furthermore, we identify that YTHDC1 acts as the fulcrum to recruit the splicing factor SRSF3 and facilitate the m^6^A-YTHDC1-SRSF3 complex-mediated RBM4 exon inclusion (Figs. 4g-h and 3h–i, Supplementary Fig. S7c–d). Whereas nuclear translocation of AURKA disrupts the binding of SRSF3 to YTHDC1, thus inhibiting RBM4 splicing towards RBM4-FL (Figs. 4g-h and 3j–l, Supplementary Fig. S8a–c). In turn, YTHDC1 recruitment of hnRNP K is bridged by nuclear AURKA, finally leading to an m^6^A-YTHDC1-hnRNP K-dependent exon skipping to produce RBM4-S (Figs. 5g–h and 5b). Together, we unveil that m^6^A recognition of YTHDC1 is required for oncogenic splicing of the tumor suppressor RBM4 provoked by nuclear AURKA.

We demonstrate that the tumor suppressor RBM4 itself, one of the critical splicing regulators, can be alternatively spliced too. With exon 3 skipping, RBM4 is spliced from RBM4-FL to RBM4-S that is translated into a short protein isoform containing only two N-terminal RNA recognition motifs (RRM) (Fig. 1d, Supplementary Fig. S12a). We find that RBM4-S is highly accumulated in primary NSCLC specimens as compared to the adjacent normal tissues (Fig. 7a). Opposite to RBM4-FL, RBM4-S significantly promotes tumor growth both in vitro and in vivo (Fig. 2b–e, Supplementary Fig. S4a–c). Previous reports demonstrate that RBM4 facilitates tumor suppression via not only controlling target pre-mRNA alternative splicing (e.g. Bcl-x and TEAD4) but also through antagonizing oncogenic SRSF1 to inhibit mTOR activation.^19,45^ Our results reveal that RBM4-S competes with RBM4-FL to elevate SRSF1 protein levels, thus activating mTOR signaling pathway in a dose-dependent manner (Fig. 2m). In the preliminary exploration of the mechanism, we find that RBM4-S overexpression significantly inhibits the protein interaction between RBM4-FL and SRSF1 (Supplementary Fig. S5d). These results suggest that RBM4-S can reverse the antagonistic effect of RBM4-FL on SRSF1 expression, possibly because RBM4-S disrupts the binding of RBM4-FL to SRSF1. Our previous studies have shown that RBM4 inhibits SRSF1 expression at a non-transcriptional level.^19^ Since these two proteins can bind to each other,^19^ and both have the ability of nucleo-cytoplasmic shuttle,^46,47^ we hypothesize that RBM4 might affect the stability of SRSF1 protein. Therefore, RBM4-S disrupts the binding of RBM4-FL to SRSF1, which may further affect the regulation of SRSF1 protein stability by RBM4-FL. Collectively, we identify a new cancer-specific splicing event and discover that RBM4-S antagonizes the function of RBM4-FL to promote tumor progression.

Therapeutically, we identify small molecules that block AURKA nuclear translocation by screening 6 drug libraries with 2216 drugs. Two chemicals, JNJ and PHA, are verified to prevent AURKA from entering the nucleus (Fig. 6a). Although PHA has been identified as an Aurora kinase inhibitor with potential anti-tumor activities,^48^ and our data also support that JNJ and PHA inhibit AURKA kinase activity (Supplementary Fig. S10a). In fact, we have confirmed that targeting AURKA kinase activity fails to disrupt the binding of AURKA to YTHDC1 and has no effect on the RBM4 splicing switch (Fig. 4e, Supplementary Fig. S2g). These results suggest that we should focus on the novel inhibition function of AURKA nuclear translocation by PHA and JNJ. Additional data verify that both JNJ and PHA treatment inhibit AURKA nuclear translocation and enhance the exon inclusion of RBM4 in lung cancer cells (Fig. 6b–c). Furthermore, nuclear AURKA accumulation abolishes RBM4 exon inclusion induced by JNJ and PHA treatment, suggesting that JNJ and PHA promote RBM4 exon inclusion through blocking AURKA nuclear translocation (Fig. 6d). Not surprisingly, the combination of AURKA kinase inhibitor (VX680) and AURKA nuclear localization inhibitors (JNJ or PHA) produces an additive tumor growth-suppressing effect (Fig. 6e–g). Overall, the discovery of AURKA nuclear translocation inhibitors provides a novel therapeutic route for future cancer treatment.

In summary, our work identifies m^6^A reader YTHDC1 as the fulcrum of tumor suppressor RBM4 oncogenic switch and reveals a previously unappreciated role of nuclear AURKA in regulating RNA splicing. Importantly, YTHDC1-directed RBM4 splicing is governed by AURKA accumulated in the nucleus of cancer cells, therefore targeting AURKA with its nuclear translocation inhibitors effectively suppresses the oncogenic switch of RBM4 and tumor progression. Overall, our results elucidate a novel mechanism of how nuclear AURKA regulates m^6^A reader YTHDC1-mediated RBM4 aberrant splicing, and provide therapeutic opportunities for lung cancer by targeting nuclear translocation of AURKA.

## Materials and methods

### Cell culture and chemicals

The human lung cancer cell lines (A549, NCI-H460, H358, and H1299), human normal bronchial epithelial cell line HBE, breast cancer cell line MDA-MB-231, liver cancer cell line HepG2 and human embryonic kidney HEK-293T cell line were obtained from the American Type Culture Collection (ATCC). These cell lines were authenticated at ATCC before purchase by standard short tandem repeat DNA-typing methodology. Each cell line was cultured in a standard medium as recommended by ATCC. All cells were incubated at 37 °C in a humidified incubator containing 5% CO_2_.

Doxycycline was purchased from Clonetech. MLN8237, VX680, JNJ-26854165, and PHA-680632 were purchased from Selleck Chemicals. AKI603 was designed and synthesized by our laboratory.^49^ Nocodazole, puromycin and MG132 were purchased from Selleck.

The natural product screening library and Autophagy library were purchased from Selleck Chemicals. International drug collection, US drug collection, Epigenetic library, and Stemness library were purchased from Topscience.

### Clinical samples

Eight pairs of lung cancer specimens and adjacent normal tissues were obtained from patients undergoing surgery, following informed consent from patients and approved by the Institutional Ethics Review Board of the First Affiliated Hospital of Dalian Medical University (PJ-KS-KY-2021-94). In addition, we also obtained primary human lung tumor tissue microarrays (HLugS120CS01) from Outdo Biotech (Shanghai, China), containing 60 lung cancer and 60 corresponding adjacent normal samples.

### Analysis of tumor growth

Female BALB/C-nu mice (6 weeks old) were subcutaneously inoculated with equal amounts (2 × 10^6^/100 μl in PBS) of A549-Vector, RBM4-FL and RBM4-S cells. Tumor formation was monitored for 34 days.

Female BALB/C-nu mice (6 weeks old) were subcutaneously inoculated with equal amounts (6 × 10^6^/100 μl in PBS containing 50% Matrigel) of NCI-H460 cells. When tumor’s volume reached more than 100 mm^3^, the animals were randomized into four treatment groups and treated as follows: PBS, VX680 (20 mg/kg/d), PHA (40 mg/kg/d) combined with VX680 (20 mg/kg/d), JNJ (20 mg/kg/d) combined with VX680 (20 mg/kg/d). The agents used in this study were administered by gavage and the treatment continued for 10 days. The tumor volumes were determined using calipers to measure the longest (length) and shortest (diameter) every three days and were calculated according to the standard formula (length × diameter^2^ × 0.5).

All animal studies were approved by the Institute Animal Care and Use Committee of Dalian Medical University (No. AAE18017), and carried out in accordance with established institutional guidelines and approved protocols.

### RNA-seq analysis

Total RNA was extracted from DOX-induced AURKA knockdown NCI-H460 cells by using Trizol reagent (Life technologies, 15596026). cDNA library preparation for total RNA was performed with the Illumina TruSeq Stranded Total RNA with Ribo-Zero Globin kit (Illumina, Inc., San Diego, CA). The paired-end reads were generated by the Illumina Hi-Seq 2000 platform and mapped to the human genome. Changes in splicing isoforms were analyzed by MISO pipeline.

### Plasmid construction and transfection

The shRNA fragment targeting AURKA was inserted into inducible pLKO-Tet-On vector.^50^ To construct the RBM4 minigene reporter, we used PCR reactions to amplify a fragment containing exon 2, part of intron 2, exon 3, part of intron 3 and exon 4 of RBM4, and ligated this fragment to the pCDNA3.1(+) and pLVX-DsRed-Monomer-N1 vectors. RBM4 minigene reporters containing mutations were obtained from wild-type RBM4 minigene reporter by performing PCR reactions and Dpn I enzyme digestion. Full-length and truncated RBM4 fragments were cloned from human genome cDNA and ligated into pLVX-DsRed-Monomer-N1 vector (Clontech) in a frame with Flag tag. The pLVX-AURKA-HA plasmid, GST-fused AURKA full-length (1–403 aa) and truncations (1–333 aa, 1–283 aa, 1–233 aa, 1–183 aa, 1–131 aa), pLVX-AURKA-EGFP plasmid, pLVX-AURKA-NLS plasmid and pLVX-AURKA-NES plasmid were kindly provided by Dr. Fei-Meng Zheng (Sun Yat-Sen University Cancer Center, Guangzhou, China).^15^ Additional AURKA truncation 333–403 aa was cloned from AURKA full-length and ligated into GST vector. pb533a-flag-YTHDC1 and pCMV-myc-YTHDC1-DM plasmids were kindly provided by Professor Yun-Gui Yang (Beijing Institute of Genomics, Chinese Academy of Sciences, Beijing, China).^12^ PCDH-Flag-YTHDC1-WT and PCDH-Flag-YTHDC1-DM plasmids were cloned from pb533a-flag-YTHDC1 and pCMV-myc-YTHDC1-DM, respectively. PZW2C-CGG1 and PGZ3-CGG1 were kindly provided by Professor Yang Wang (Dalian Medical University, Dalian, China).^19,51^ SRSF1 with HA tag was generated by PCR amplification and subcloned into PCDH-CMV-MCS-EF1-Puro vector using NheI/NotI restriction enzyme sites. SRSF3 with an N-terminal Flag epitope was generated by PCR amplification and subcloned into PCDH-CMV-MCS-EF1-Puro vector using NheI/NotI restriction enzyme sites. The shRNA targeting SRSF3 was inserted into pLKO.1-puro by AgeI and EcoRI restriction sites. The Fidelity of all vectors was confirmed by DNA sequencing. Expression plasmids were transfected into cells using lipofectamine 2000 or lipofectamine 3000 (Invitrogen) according to the manufacturer’s instructions. All primers used in this study were listed in Supplementary Table 6.

### RNA interference

All the RNA interference fragments were synthesized and purchased from GenePharma. The target sequences of AURKA, RBM4-FL, hnRNP A1, hnRNP L, hnRNP A2B1, hnRNP U, hnRNP K, METTL3, YTHDC1, SRSF3, hnRNP C, IGF2BP3, and control siRNAs were listed in Supplementary Table 7.

### Lentivirus preparation

HEK-293T cells were used for packaging lentivirus with the 2nd generation packaging system plasmid psPAX2 (Addgene) and pMD2.G (Addgene). Lentiviruses were concentrated by ultracentrifugation, and viral titer determined by serial dilutions. For infection with the lentivirus, infected cells were selected with Puromycin (2 μg/ml).

### RNA extraction and reverse transcription-PCR (RT-PCR)

Total RNA was extracted by using TRIzol reagent (Life technologies, 15596026). cDNA was generated by using EasyScript One-Step gDNA Removal and cDNA Synthesis SuperMix Kit (TransGen Biotech, #AE311-03) according to the manufacturer’s instructions. PCR amplification was performed in a 20 μl reaction system by using 2×EasyTaq PCR SuperMix (TransGen Biotech, AS111). ACTB was used as the internal control. All primers used in RT-PCR were listed in Supplementary Table 8.

### Real-time quantitative PCR (RT-qPCR)

Quantitative PCR was performed with SYBR green (HiScript® III RT SuperMix for qPCR, Vazyme) at Agilent Mx3005P real-time PCR system (Agilent Technologies). Expression analysis in different cell types was performed using specific primers for each gene. All primers used in RT-qPCR were listed in Supplementary Table 8.

Analysis of RNA immunoprecipitation (RIP), RNA fluorescent in situ hybridization (FISH), RNA pull-down, methylated RNA-Immunoprecipitation (MeRIP), immunofluorescence (IF) analysis, western blot analysis, co-immunoprecipitation (Co-IP) analysis, cytoplasmic/nuclear protein extraction, immunohistochemistry (IHC) assay and H-score analysis, colony formation assay, CCK8 proliferative assay, and statistical analysis

## Supplementary information

Supplementary_Materials

Supplementary figure 1

Supplementary figure 2

Supplementary figure 3

Supplementary figure 4

Supplementary figure 5

Supplementary figure 6

Supplementary figure 7

Supplementary figure 8

Supplementary figure 9

Supplementary figure 10

Supplementary figure 11

Supplementary figure 12

Supplementary Table 1 SILAC

Supplementary Table 2 RNAXXXseq

Supplementary Table 3 UniProt Keywords

Supplementary Table 4 AURKA-RIP-seq

Supplementary Table 5 spliceosome activity correlation analysis

Supplementary Table 6 Plasmid primers

Supplementary Table 7 siRNA

Supplementary Table 8 RT-PCR and qPCR primers

Supplementary Table 9 PCR primers for in vitro transcription

## Data Availability

All data are available within the article and its Supplementary Information.
