# Supplementary material for: Nuclear Aurora kinase A switches m6A reader YTHDC1 to enhance an oncogenic RNA splicing of tumor suppressor RBM4
Source: Signal Transduct Target Ther. 2022 Apr 1;7:97. doi: 10.1038/s41392-022-00905-3 (PMC8971511; doi:10.1038/s41392-022-00905-3)
Supplement: Supplementary file 1 — Supplementary_Materials [file 41392_2022_905_MOESM1_ESM.docx]

Supplementary Materials for

Nuclear Aurora kinase A switches m^6^A reader YTHDC1 to enhance an oncogenic RNA splicing of tumor suppressor RBM4

SiSi Li^#^, YangFan Qi^#^, JiaChuan Yu^#^, YuChao Hao^#^, Bin He^#^, MengJuan Zhang, ZhenWei Dai, TongHui Jiang, SuYi Li, Fang Huang, Ning Chen, Jing Wang, MengYing Yang, DaPeng Liang, Fan An, JinYao Zhao, WenJun Fan, YuJia Pan, ZiQian Deng, YuanYuan Luo, Tao Guo, Fei Peng, ZhiJie Hou, ChunLi Wang, FeiMeng Zheng, LingZhi Xu, Jie Xu, QingPing Wen, BiLian Jin^*^, Yang Wang^*^, Quentin Liu^*^

Correspondence to: [liuq9@mail.sysu.edu.cn](mailto:liuq9@mail.sysu.edu.cn)

**This PDF file includes:**

Materials and Methods

Figures. S1 to S12

Materials and Methods

*RNA immunoprecipitation (RIP)*

Cells were lysed in ice-cold GLB buffer (10 mM Tris-HCL (PH 7.5), 10 mM NaCl, 10 mM EDTA, 0.5% TritonX-100) freshly supplemented with indicated concentrations of protease inhibitor Cocktail (MCE, HY-K0011), PMSF, DTT and RNase OUT (Invitrogen, 10777019). Lysates were mixed gently and incubated for 30 minutes on ice, then centrifuged at 12000g for 15 minutes at 4°C. Ribonucleoprotein particle-enriched lysates were incubated with protein A/G-plus agarose beads (Roche, #11719233001) together with primary antibody or normal IgG for 4 hours at 4°C. Extensive washing was performed with GLB+ buffer (50 ml GLB supplemented with 1.75 ml 4 M NaCl) containing tRNA (Invitrogen, AM7119). RNA was isolated, purified with RNeasy Kit (Qiagen) and subjected to RT-PCR analysis. Protein was eluted with loading buffer by boiling 5 minutes at 100℃ and subjected to immunoblot analysis.

*RNA fluorescent in situ hybridization (FISH)*

5’digoxigenin (DIG) labeled locked nucleic acid FISH probes were generated by *in vitro* transcription (MEGAscript™ T7 Transcription Kit, Invitrogen, AM1333). Cell cultures were fixed in buffered 3.6% PFA with 10% acetic acid (in 1×PBS, pH 7.2-7.4), permeabilized with 0.2% TritonX-100 with 2 mM VRC (1:200) for 5 minutes at room temperature. Rehydrolized in 50% formamide with 2×SSC for 10 minutes at room temperature, then pre-hybridizated at 55°C for 2 hours with hybridization buffer without dextran sulfate. Incubated with preheated probes (90–95°C) at 55°C overnight in 50% formamide and hybridization buffer containing 30-60 ng of probe. Following hybridization, cells were washed twice in 0.1×SSC with 0.1% SDS and 50% formamide (pH 7.2) for 30 minutes each at 55°C, and once in 2×SSC with 50% formamide (pH 7.2) for 15 minutes at 55°C. After blocking with blocking buffer at 37°C for 30 minutes, an unconjugated sheep anti-Digoxigenin antibody (Roche Diagnostics, 1:100) and AURKA antibody (Abcam, ab1287, 1:100) were used followed by the appropriate secondary antibody (Life technologies corporation, A21206, A21436, 1:100). Cells then underwent a series of 5 minutes washes with 2×SSC with 8% formamide, and were mounted onto slides with ProLong Antifade Gold mounting media with DAPI (Sigma Aldrich, 1 μg/ml). Images were acquired using a confocal microscope (Leica). All primers used for *in vitro* transcription were listed in Supplementary Table 9.

*RNA pull-down*

Biotinylated RNA transcripts were *in vitro* transcripted from PCR fragments or minigene reporters with T7 promoter using MEGAscript T7 kit (Ambion, AM1333) with biotin-16-UTP (Ambion, AM8452). Cells were lysed in lysis buffer (1% NP-40 in DEPC-dH_2_O prepared PBS) freshly supplemented with indicated concentrations of protease inhibitor Cocktail for 30 minutes on ice. Recovered the secondary structure of biotinylated RNA with binding buffer (10 mM HEPES, 50 mM KCl, 10% glycerol, 1 mM EDTA, 1 mM DTT, 0.5% TritonX-100). The lysate was then pre-cleared using streptavidin agarose beads (Thermo, 15942-050) for 30 minutes. Mixed 30 ul RNA, 50 ul cell lysate, 3 ul cocktail, 1 ul RNase OUT (Invitrogen, 10777019), 2 ul Yeast tRNA (Invitrogen, AM7119) and added RNase free dH_2_O up to 300uL in an RNase free Ep tube. Rotated at room temperature gently for 1 hour. After activating the streptavidin agarose beads, blocking the beads in blocking buffer (1 ml binding buffer, 25 ul 20% BSA, 5 ul Yeast tRNA) for 30 minutes. Then transferred the mixture to the blocked beads by gently rotating at room temperature for 30 minutes in the rotator. Prepared 100~150 mM NaCl binding buffer to wash beads for 7 times. Next, the beads were boiled at 100℃ with 50 ul 2*loading buffer and then subjected to western blot analysis. All primers used for *in vitro* transcription were listed in Supplementary Table 9.

*Methylated RNA Immunoprecipitation (MeRIP)*

Total RNAs were extracted with TRIzol reagent (Life technologies, 15596026). Intact mRNAs were highly purified from total RNAs using Dynabeads mRNA purification kit (Ambion, 61006). For m^6^A-RIP-PCR, the procedure was modified from the previously reported methods ^1^. In brief, purified mRNAs were digested by DNase I (NEB, M0303) and then fragmented into around 300-nt fragments by incubation at 94°C for 1 minute with RNA Fragmentation Reagents (Ambion, AM8740), followed by standard ethanol precipitation and collection. Anti-m^6^A polyclonal antibody (10 μg antibody for 5 μg mRNAs; Synaptic Systems, 202003) was incubated with 40 μl Dynabeads Protein A for Immunoprecipitation (Thermo, 10001D) in IPP buffer (150 mM NaCl, 0.1% NP-40, 10 mM Tris-HCl, pH 7.4) for 1 hour at room temperature. Then the mRNAs (5 μg) were incubated with the prepared antibody-beads mixture for 4 hours at 4°C. After washing, bound RNAs were extracted by Acid Phenol: Chloroform (pH 4.3-4.7) (Ambion, AM9720). The mRNAs pulled down by anti-m^6^A antibody were then reversely transcribed and amplified. The enrichment of m^6^A was quantified using PCR. The sequences of PCR primers are listed in Supplementary Table 8.

*Immunofluorescence (IF) analysis*

Cells were grown on glass cover lips in a proper confluence for 24 hours. After fixed in 4% (v/v) formaldehyde/PBS, cells were permeabilized (0.5% TritonX-100/PBS) and blocked for 1 hour in 5% BSA/PBS. IF was performed at room temperature using antibodies. Alexa-conjugated anti-IgG antibodies (Invitrogen Corp, 1:200) were used for secondary detection. DAPI (Sigma Aldrich, 1 μg/ml) was used for nuclear staining. Images were acquired using a confocal microscope (Leica).

*Western blot analysis*

Samples were lysed on ice in RIPA buffer (50 mM Tris [pH 8.0], 150 mM sodium chloride, 0.5% sodium deoxycholate, 0.1% SDS, and 1% NP-40) supplemented with protease inhibitors. The protein concentration was determined by the Coomassie brilliant blue dye method. In all, equal amounts of protein per lane were run in 10% to 15% SDS–PAGE gels and subsequently transferred to a nitrocellulose membrane (Millipore) via submerged transfer. After blocking the membrane with 5% fat-free milk or 3% BSA in TBST at room temperature for 1 hour, the membrane was incubated overnight at 4°C with various primary antibodies. After incubation with peroxidase-conjugated secondary antibodies for 1 hour at room temperature, the signals were visualized using an enhanced chemiluminescence Western blot detection kit (K-12045-D50; Apgbio, Beijing, China) according to the manufacturer’s instructions. The blots were developed using the Bio-Rad Molecular Imager instrument (Bio-Rad, USA). The information of antibodies were listed as follows: AURKA (Upstate, 07-648), P-AURKA (Cell Signaling Technology, D13A11), GAPDH (KANGCHEN, KC-5G4), Alpha Tubulin (Proteintech, 11224-1-AP), hnRNP K (Cell Signaling Technology, 4675S), YTHDC1 (Abcam, ab122340), m^6^A (Synaptic System, 202003), METTL3 (Proteintech, 15073-1-AP), SRSF3 (MBL, RN080PW), AURKA (Sigma, a1231), RBM4 (ATLAS ANTIBODIES, HPA042174), HA (Sigma, H6908), Flag (Sigma, F1804), GST (Thermo, RB231555), LaminB (Proteintech, 66095-1-lg), p70 S6 kinase (Proteintech, 14485-1-AP), phosphor-p70 S6 kinase (Cell Signaling Technology, #9234), phosphor-4E-BP1 (Cell Signaling Technology, #9455), 4E-BP1 (Cell Signaling Technology, #9644), Goat anti-Mouse IgG (HRP conjugated) (Thermo-Pierce, 31430), Goat anti-Rabbit IgG (HRP conjugated) (Thermo-Pierce, 31460). SRSF1 antibody was purchased from SCBT.

*Co-immunoprecipitation (Co-IP) analysis*

Protein lysates (500-1000 μg) prepared from cultured cells. Immuno-complex pull-down was achieved via overnight incubation of protein lysates with relevant antibodies bound to Protein A-G agarose or with Glutathione Sepharose beads (GE Healthcare) alone at 4°C. After careful washing, loading buffer was added, and the samples were boiled at 100°C for 10 minutes. Co-immunoprecipitated proteins were then subjected to western blot as described above.

*Cytoplasmic/nuclear protein extraction*

Cytoplasmic extracts were prepared by resuspending the cell pellets in hypotonic buffer (20 mM Tris-HCl, PH 7.4, 10 mM NaCl, 3 mM MgCl_2_) supplemented with protease inhibitors and then were incubated on ice for 20 minutes. Next, 15μl 10% NP-40 was added, after 10 seconds’ reaction, the lysates were centrifuged for 10 minutes at 3000 rpm at 4°C. The supernatants were transferred to new Eppendorf tubes. Then the residual samples were washed with hypotonic buffer for 4 times and the supernatants were discarded thoroughly. To obtain nuclear extracts, the nuclear fraction was treated with nuclear extraction buffer (100 mM Tris-HCl, PH 7.4, 2 mM Na_3_VO_4_, 100 mM NaCl, 1% TritonX-100, 1 mM EGTA, 0.1% SDS, 1 mM NaF, 20 mM Na_4_P_2_O_7_). After pipetting up and down for 30 minutes at 4°C, the lysates were centrifuged at 14000 g for 30 minutes at 4°C. Supernatants after this spin contained the nuclear protein preparation.

*Immunohistochemistry (IHC) assay and H-score analysis*

Briefly, the sections were deparaffinnized in xylene, rehydrated and incubated in 3% (v/v) hydrogen peroxide (Sigma-Aldrich, 323381) for 10 minutes to remove the activities of endogenous peroxidases. Antigenic retrieval was processed with sodium citrate. Then the sections were incubated overnight at 4°C with the indicated antibodies in a moist chamber. After an incubation with the universal secondary antibody (Solution B; SP-9000, ZSGB-BIO, China), the slides were incubated with streptavidin which was labeled by the horse radish peroxidase (Solution C; SP-9000, ZSGB-BIO, China) for 15 minutes at room temperature. The reaction products were visualized by staining with DAB (3, 3’-Diaminobenzidine; ZSGB-BIO, China). Finally, the sections were counterstained with hematoxylin, dehydrated and mounted. Negative controls were employed by replacing the primary antibody with the non-immune serum immunoglobulin.

The IHC staining was quantified by the H-score system, which incorporates the intensity of the staining and the percentage of positive cells. The relative intensity of staining was defined as negative (0), weak (1+), distinct (2+) and strong (3+) ^2^. The final score was the sum of the relative intensity of staining multiplied by the percentage of positive cells. H-score analysis was performed independently by two experienced pathologists who were blinded to the final clinical diagnosis of all cases studied.

*Colony formation assay*

Log-phased cells (2×10^3^ cells per dish) were plated into 6-well plate and cultured at 37°C equipped with 5% CO_2_. Cells were fed with fresh growth medium every 2 days. After 10 days of incubation, colonies were fixed with 4% PFA, stained with crystal violet, and counted using Image J software. Each experiment was repeated three times.

*CCK8 proliferative assay*

Log-phased cells (2×10^3^ cells) were plated into 96-well plate and cultured at 37°C equipped with 5% CO_2_. 10% CCK8 (Selleck) was added to each well and cells were incubated for 4 hours. The absorbance (OD) was measured at 450 nm using a multimode plate reader (Perkin Elmer).

*Statistical analysis*

Each *in vivo* and *in vitro* experiment was performed in triplicate and repeated at least three times. Unless otherwise indicated, data were presented as means of three independent experiments. Statistical analyses were performed with SPSS software (version 16.0) or GraphPad Prism 7.0 (GraphPad Software, Inc.). Differences among variables were assessed by two-tailed unpaired Student’s t-test. A p-value less than 0.05 was considered statistically significant.

**Reference**

1. Chen T*, et al.* m(6)A RNA methylation is regulated by microRNAs and promotes reprogramming to pluripotency. *Cell Stem Cell* **16**, 289-301 (2015).

2. Yang Y*, et al.* H-score of 11β-hydroxylase and aldosterone synthase in the histopathological diagnosis of adrenocortical tumors. *Endocrine* **65**, 683-691 (2019).


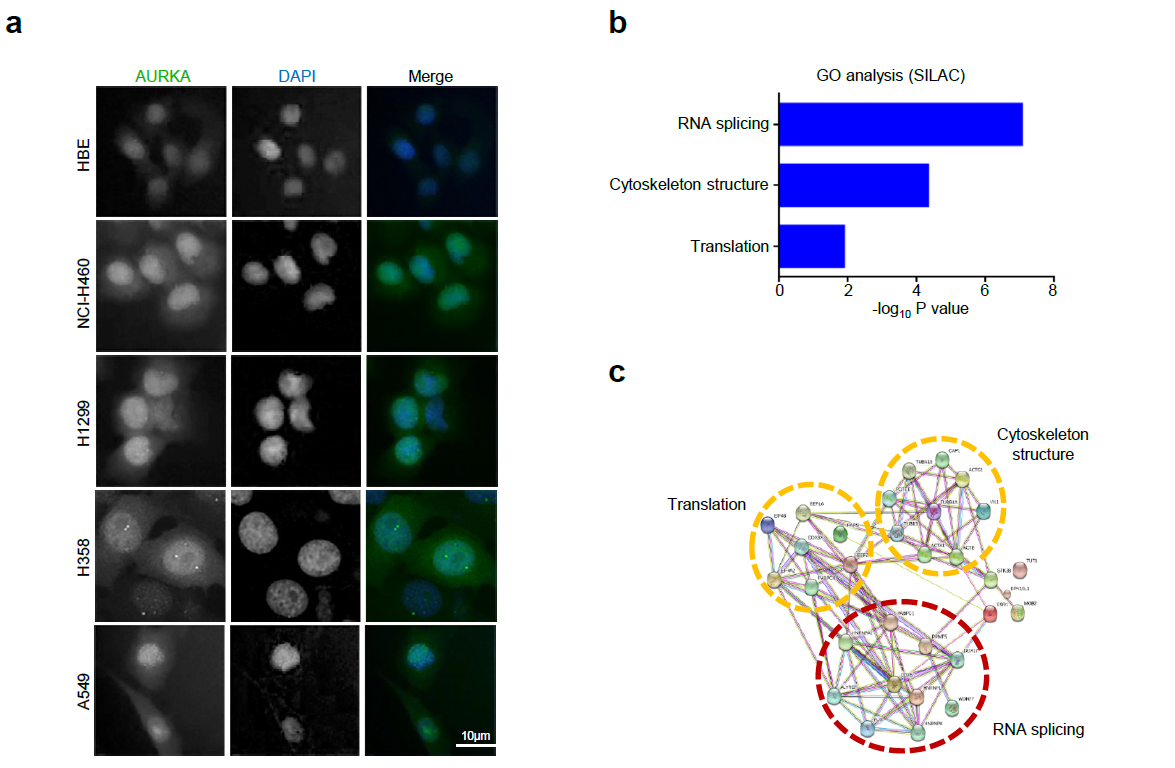


**Figure S1 Functional analysis of AURKA interacting proteins. (a)** The expression and distribution of AURKA (green) were analyzed via immunofluorescence (IF) staining in HBE, NCI-H460, H1299, H358 and A549 cells. The nuclei were stained with DAPI (blue). Scale bar, 10 μm. **(b)** Gene ontology analysis of AURKA-interacting proteins. AURKA-interacting proteins were identified using SILAC assay. **(c)** Identification of functional association network of AURKA-interacting proteins by using STRING analysis.


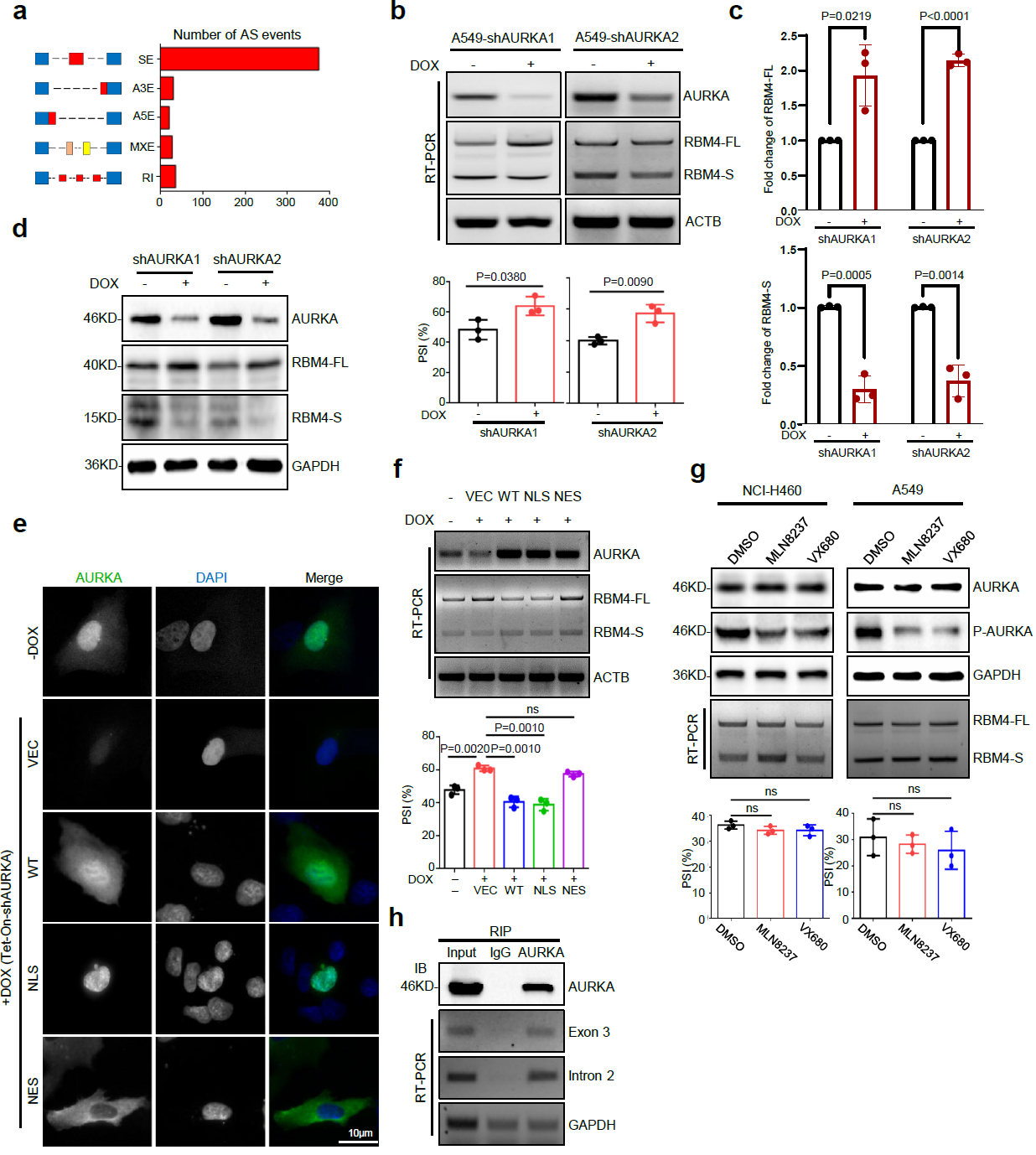


**Figure S2 Nuclear AURKA promotes RBM4 aberrant splicing in a kinase independent manner. (a)** Quantification of the different alternative splicing (AS) events affected by AURKA knockdown in NCI-H460 cells. **(b)** Validation of RBM4 splicing change by RT-PCR in DOX-induced AURKA depleted A549 cells. **(c)** RT-qPCR assay was conducted to detect RBM4-FL and RBM4-S mRNA expression levels in DOX-induced AURKA depleted A549 cells. **(d)** Validation of the protein expression of RBM4 two isoforms by western blot in DOX-induced AURKA knockdown NCI-H460 cells. **(e)** Empty vector (VEC), AURKA-WT (wild-type), AURKA-NLS (nuclear localized sequence) and AURKA-NES (nuclear export sequence) were transfected into endogenous AURKA depleted A549 cells. The localization of AURKA (green) was detected by IF assay with anti-AURKA antibody. The nuclei were stained with DAPI (blue). Scale bar, 10 μm. **(f)** RBM4 splicing change in VEC, AURKA-WT, AURKA-NLS and AURKA-NES reconstitution A549-shAURKA cells was measured by RT-PCR. **(g)** AURKA kinase inhibitors MLN8237 and VX680 were employed to treat NCI-H460 and A549 cells. The efficiency of kinase activity inhibition and the effect on RBM4 splicing were indicated by western blot and RT-PCR analysis. **(h)** Binding of RBM4 pre-mRNA with AURKA protein was detected by RIP assay in A549 cells. Data are shown as means ± SD. P values were calculated with two-tailed unpaired Student’s t-test and P < 0.05 is considered statistically significant.


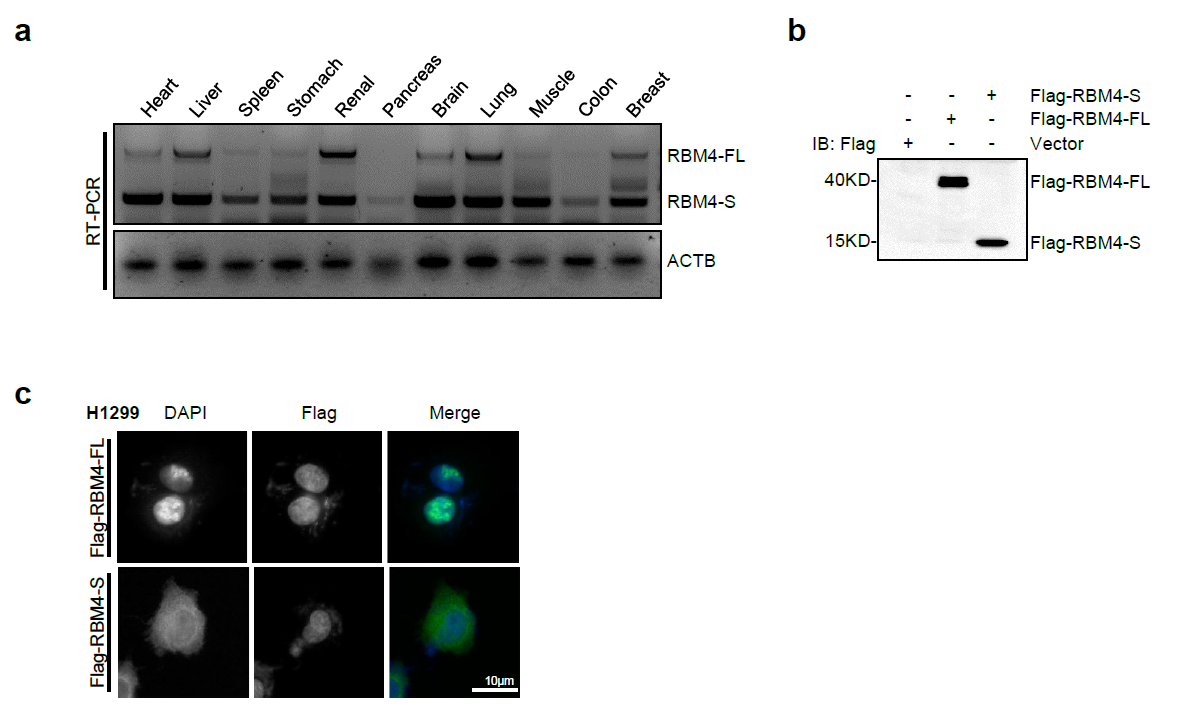


**Figure S3 The expression and localization of RBM4 two isoforms. (a)** Validation of RBM4 splicing change in different tissues of B6 mouse by RT-PCR. **(b)** Flag tagged empty vector, RBM4-FL and RBM4-S were transfected into A549 cells. Western blot assay was conducted to test the protein expression of Flag-RBM4-FL and Flag-RBM4-S by using anti-Flag antibody. **(c)** The localization of Flag-RBM4-FL and Flag-RBM4-S in H1299 cells was detected by IF assay with anti-Flag antibody (green). The nuclei were stained with DAPI (blue). Scale bar, 10 μm.


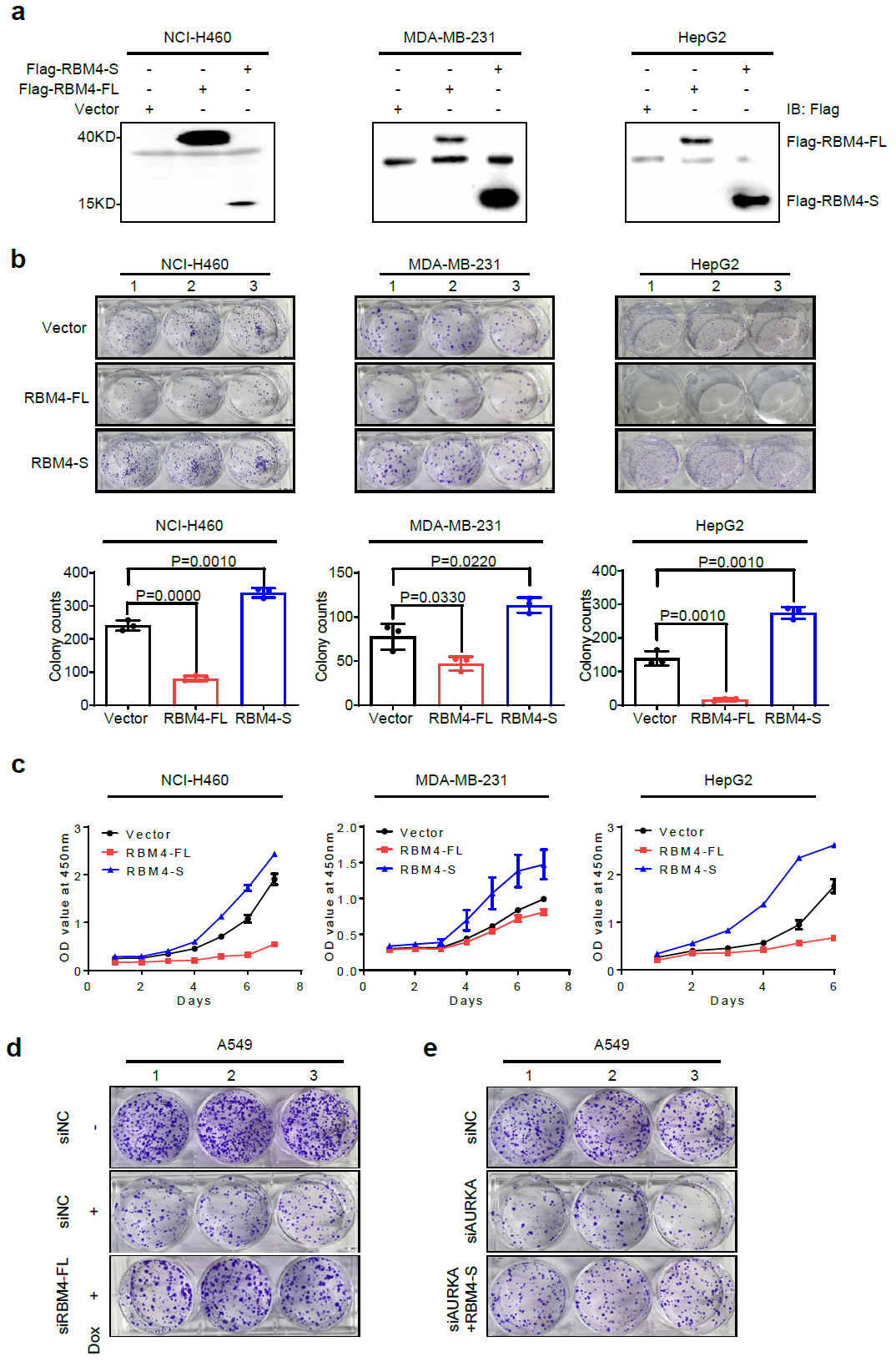


**Figure S4 Effects of RBM4 two isoforms on the proliferation of cancer cells. (a)** Flag tagged empty vector, RBM4-FL and RBM4-S were transfected into NCI-H460, MDA-MB-231 and HepG2 cells. Western blot assay was conducted to test the protein expression of Flag-RBM4-FL and Flag-RBM4-S by using anti-Flag antibody. The effects of RBM4-FL and RBM4-S on the proliferation of NCI-H460, MDA-MB-231 and HepG2 cells. The cells were stably transfected with Vector, RBM4-FL, RBM4-S and analyzed by colony formation **(b)** and cell counting kit-8 (CCK8) proliferative assays **(c)**. **(d-e)** Colony formation assay was conducted to measure the proliferation ability of different groups. Data are shown as means ± SD. P values were calculated with two-tailed unpaired Student’s t-test and P < 0.05 is considered statistically significant.


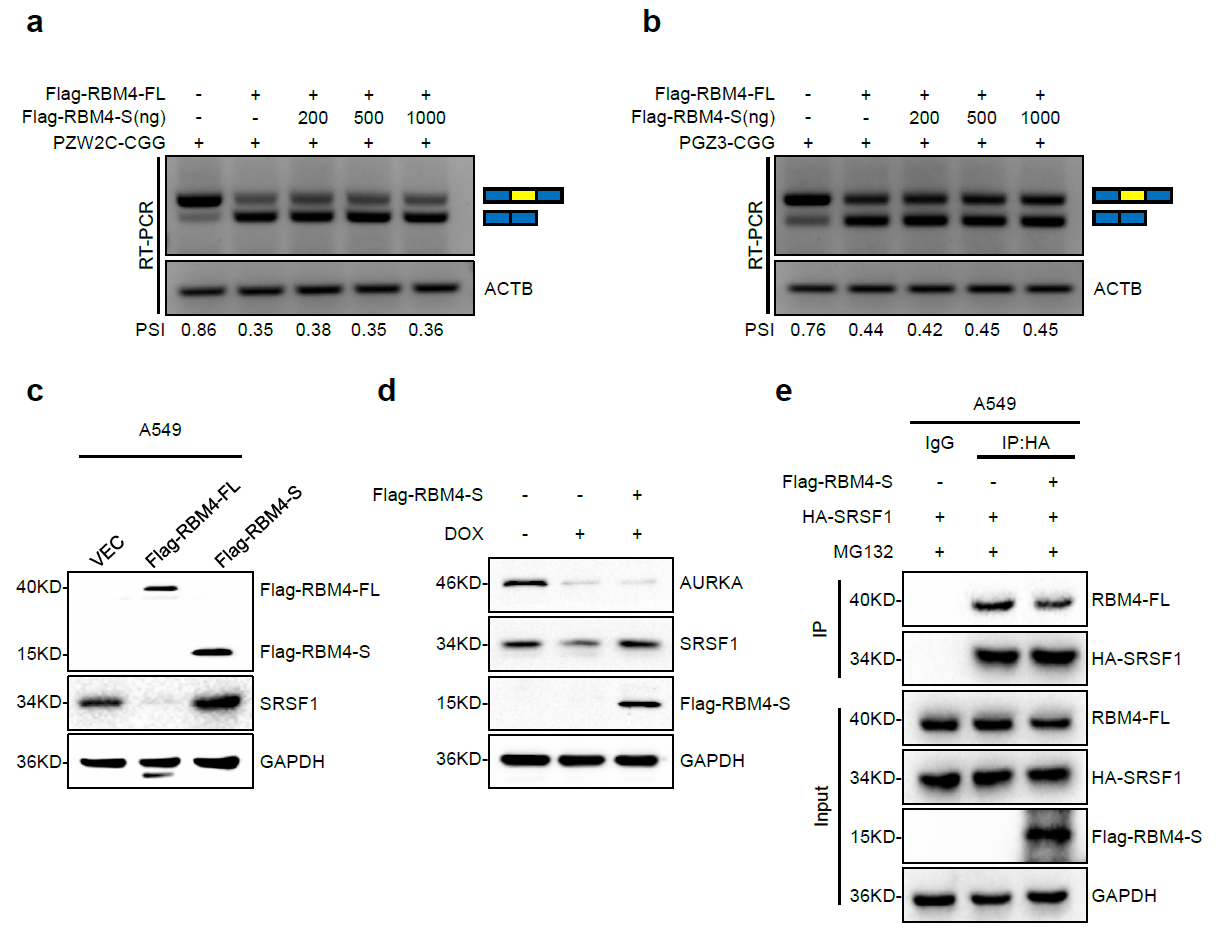


**Figure S5** **The opposite effect of RBM4-S and RBM4-FL on SRSF1 protein expression. (a-b)** Increasing amounts of Flag-RBM4-S were co-expressed with Flag-RBM4-FL and a splicing reporter containing RBM4 binding site in a cassette exon or at downstream intron in HEK-293T cells. Splicing changes were examined by RT-PCR. **(c-d)** Western blot assay was conducted to detect the expression levels of target protein in different groups. **(e)** Co-IP analysis of the protein interaction between HA-SRSF1 and RBM4-FL in NCI-H460 cells transfected with plasmid encoding Flag-RBM4-S after MG132 treatment. MG132, 20 mM.


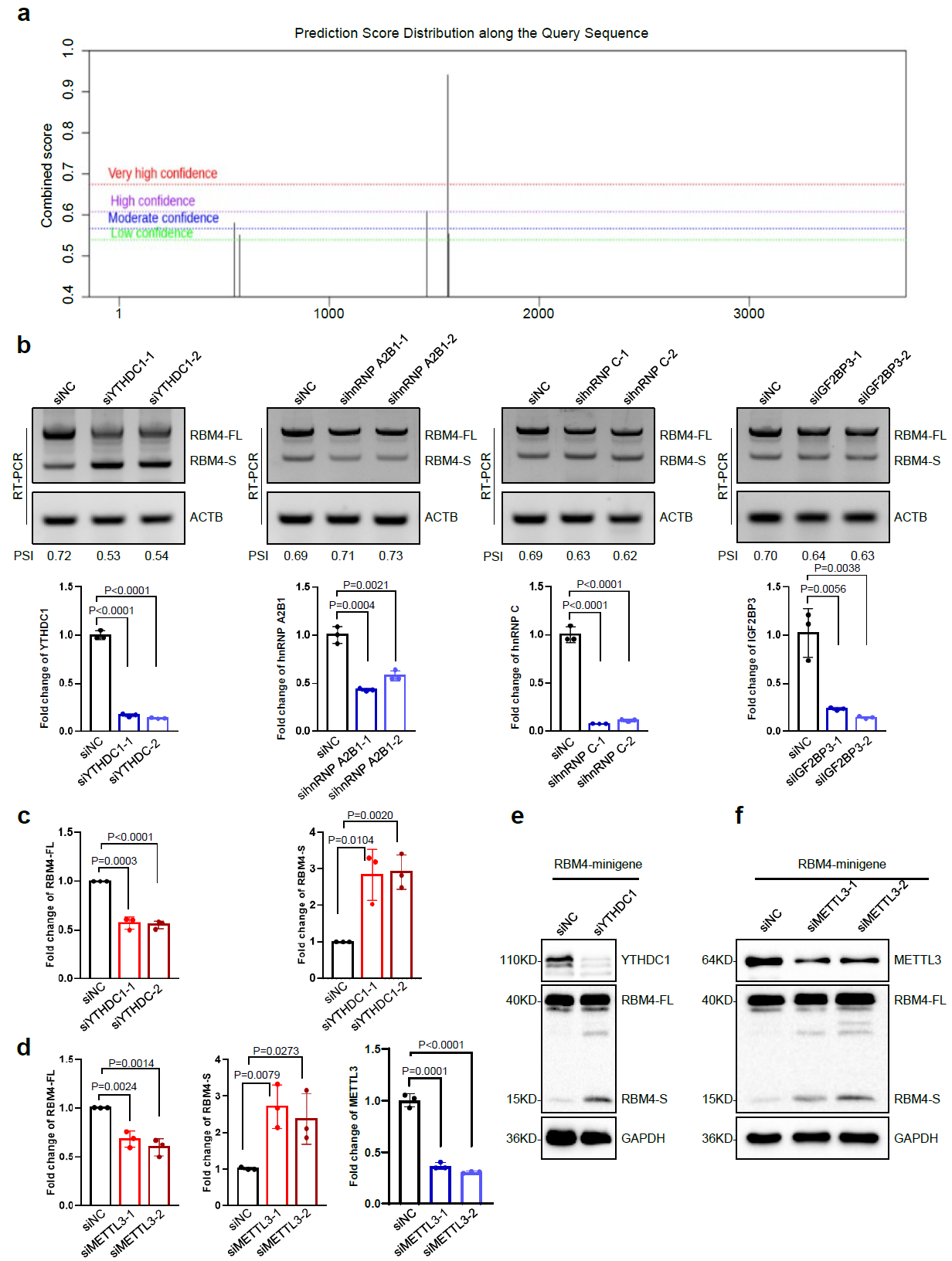


**Figure S6 m^6^A reader YTHDC1 regulates RBM4 alternative splicing. (a)** m^6^A sites of RBM4 pre-mRNA were predicted by SRAMP. **(b)** Validation of RBM4 splicing change in YTHDC1/hnRNP A2B1/hnRNP C/IGF2BP3 depleted A549 cells by RT-PCR and qPCR analysis. RT-qPCR assay was conducted to detect RBM4-FL and RBM4-S mRNA expression levels in YTHDC1 **(c)** or METTL3 **(d)** knockdown A549 cells. **(e)** RBM4 splicing reporter was overexpressed in control and YTHDC1 knockdown A549 cells. Western blot was performed to determine the splicing change of RBM4. **(f)** RBM4 splicing reporter was transiently expressed in control and METTL3 knockdown A549 cells. Western blot was conducted to assay for the splicing change of RBM4. Data are shown as means ± SD. P values were calculated with two-tailed unpaired Student’s t-test and P < 0.05 is considered statistically significant.


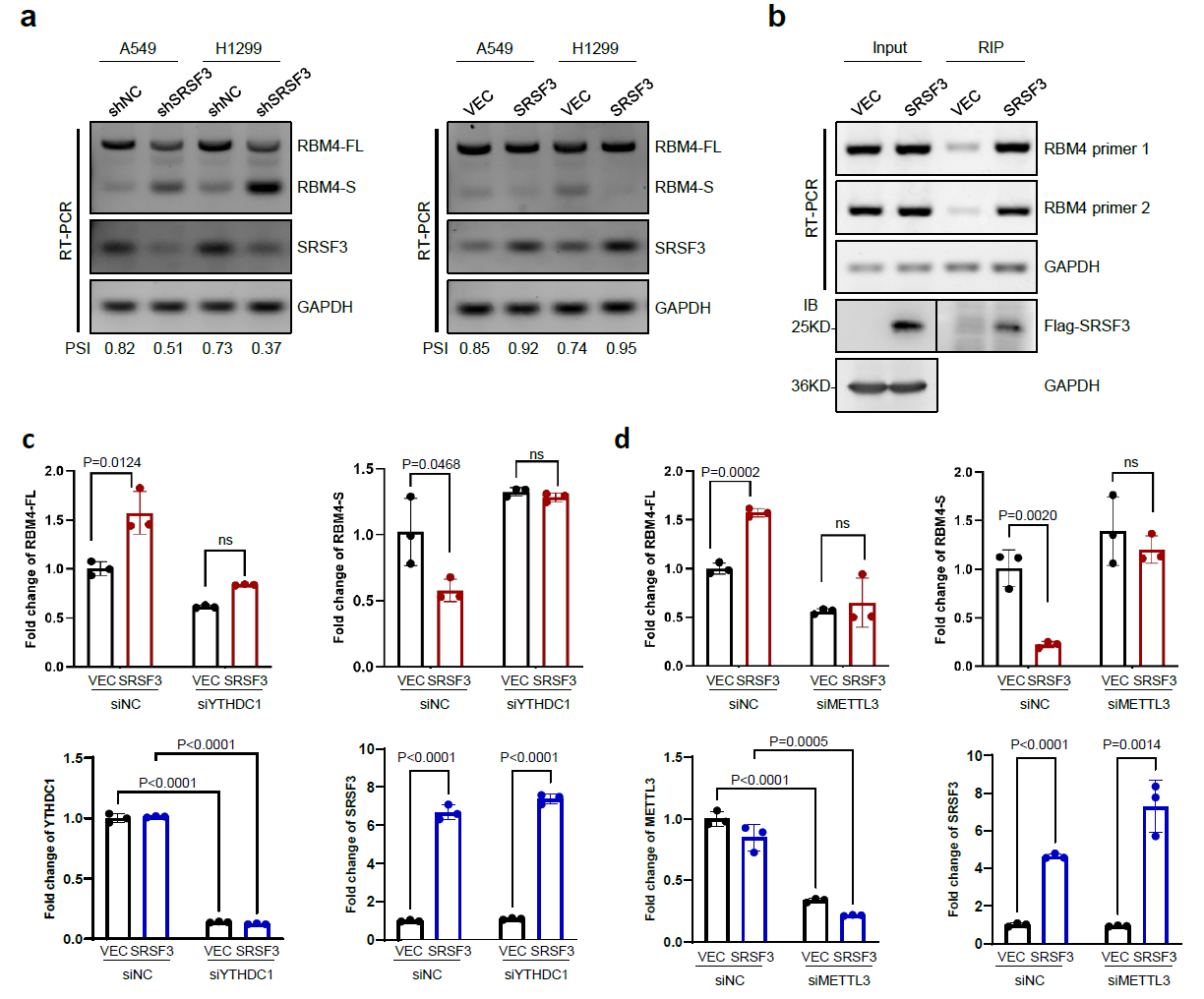


**Figure S7** **SRSF3 promotes RBM4 exon inclusion dependent on m^6^A reader YTHDC1. SRSF3 promotes RBM4 exon inclusion dependent on m^6^A reader YTHDC1. (a)** Validation of RBM4 splicing change by RT-PCR in SRSF3 knockdown and SRSF3 overexpressed A549/H1299 cells. **(b)** Binding of RBM4 pre-mRNA with SRSF3 protein was detected by RIP assay in control and SRSF3 overexpressed H1299 cells. **(c)** The level of YTHDC1 or **(d)** METTL3 was depleted in control and SRSF3 overexpressed A549 cells. Relative mRNA abundance of RBM4-FL, RBM4-S, SRSF3 and YTHDC1/METTL3 was examined by RT-qPCR. Data are shown as means ± SD. P values were calculated with two-tailed unpaired Student’s t-test and P < 0.05 is considered statistically significant.


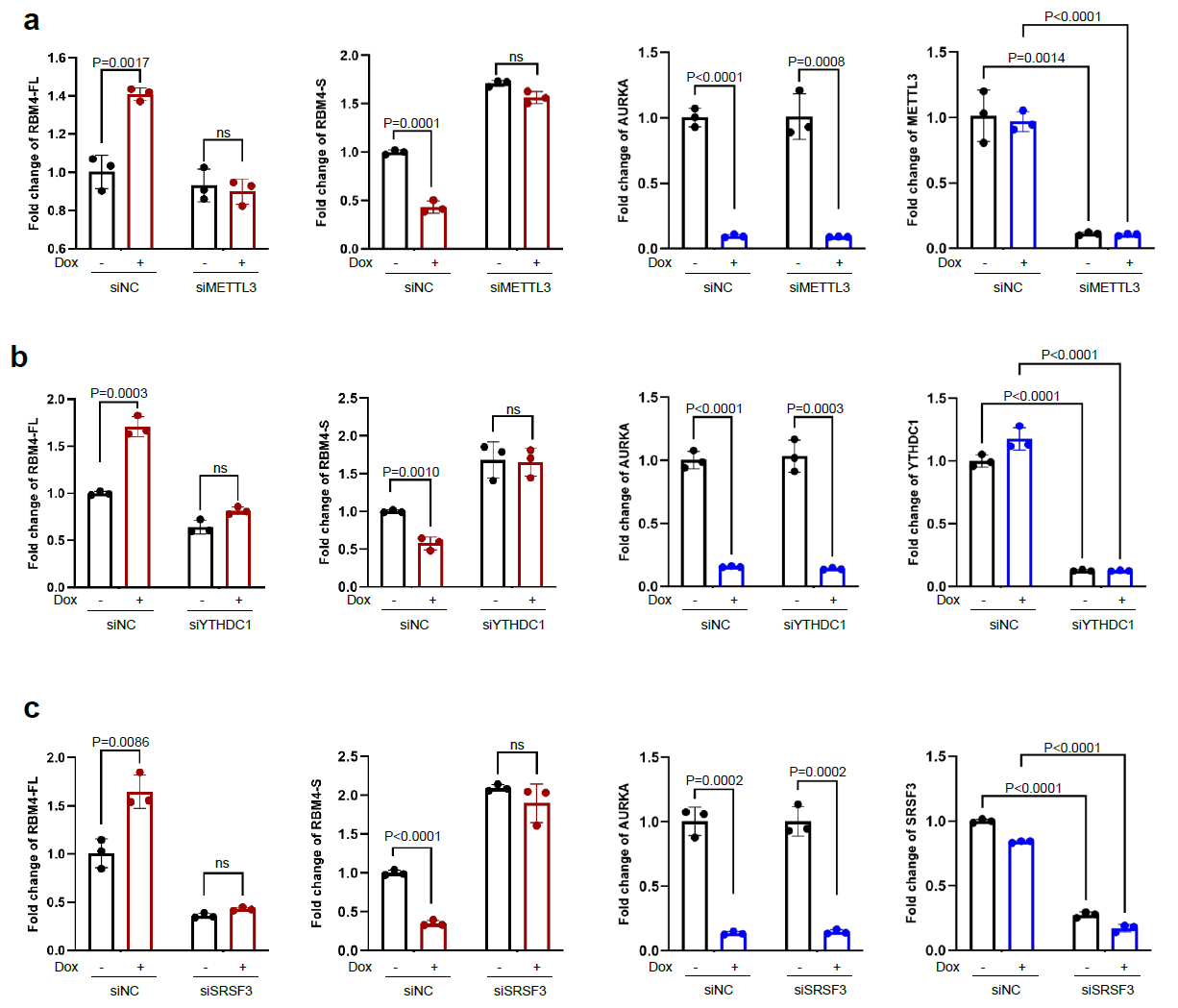


**Figure S8 AURKA inhibits m^6^A-YTHDC1-SRSF3 complex-mediated RBM4 exon inclusion.** **(a)** The level of METTL3 or **(b)** YTHDC1 or **(c)** SRSF3 was depleted in A549-Tet-On-shAURKA cells with or without DOX induction. Relative mRNA abundance of RBM4-FL, RBM4-S, AURKA and METTL3/YTHDC1/SRSF3 was examined by RT-qPCR. Data are shown as means ± SD. P values were calculated with two-tailed unpaired Student’s t-test and P < 0.05 is considered statistically significant.


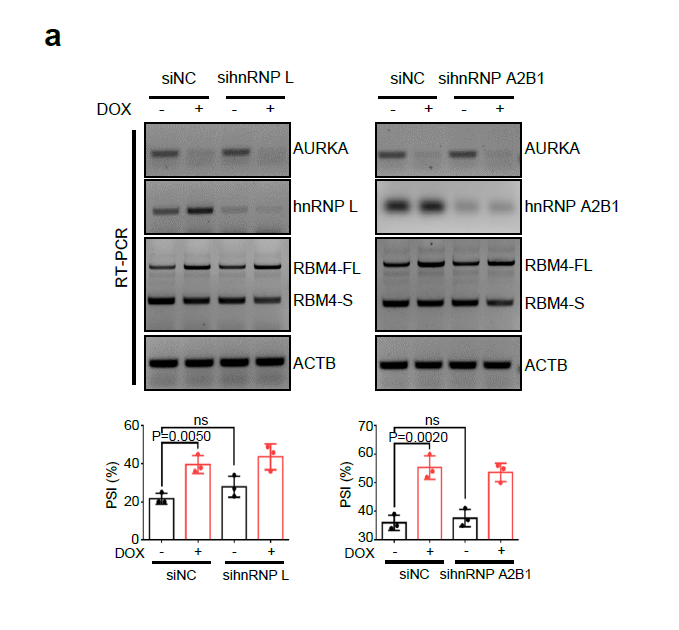


**Figure S9 hnRNP L and hnRNP A2B1 have no effect on RBM4 splicing regulation. (a)** Depletion of hnRNP L and hnRNP A2B1 in A549-Tet-On-shAURKA cells with or without DOX induction. Relative mRNA abundance of AURKA, hnRNPs and RBM4 splicing was examined by RT-PCR. Data are shown as means ± SD. P values were calculated with two-tailed unpaired Student’s t-test and P < 0.05 is considered statistically significant.


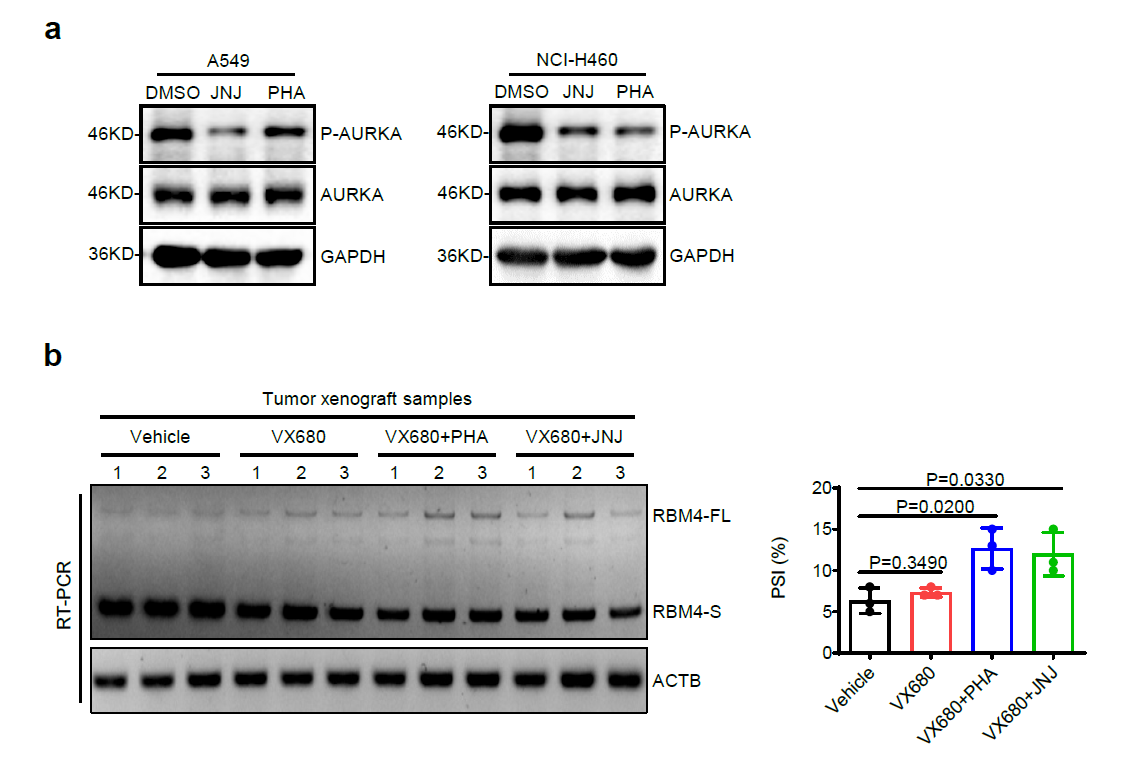


**Figure S10 Splicing changes of RBM4 in control group, VX680 alone group and drug combination group. (a)** P-AURKA and AURKA protein levels in A549 and NCI-H460 cells treated with DMSO, JNJ and PHA were tested by western blot. **(b)** RBM4 splicing change in different groups of xenograft tumors was detected by RT-PCR. Data are shown as means ± SD. P values were calculated with two-tailed unpaired Student’s t-test and P < 0.05 is considered statistically significant.


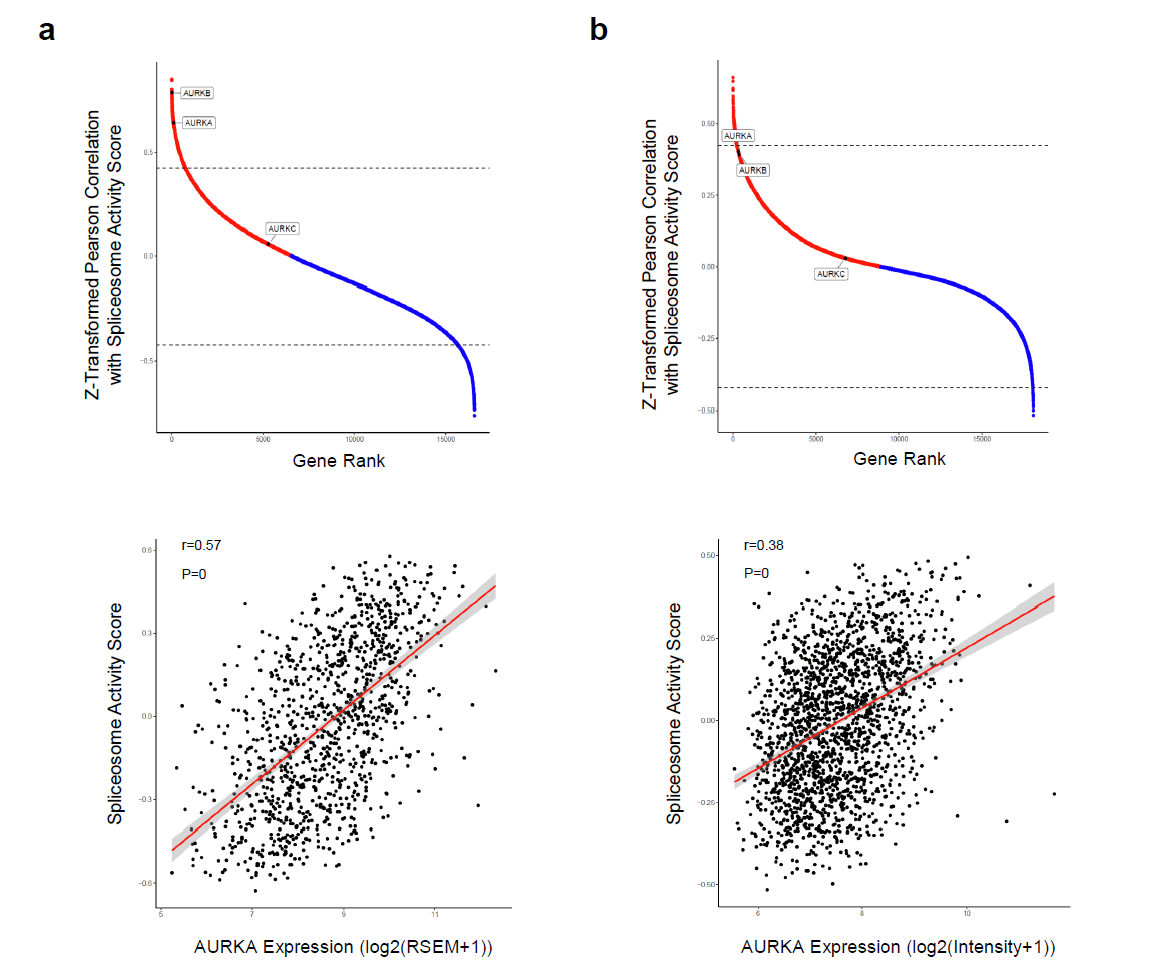


**Figure S11 Correlation analysis of gene expression and spliceosome activity score. (a)** The correlation analysis about whole genome gene expression (clinical breast cancer samples from TCGA) and spliceosome activity score. The correlation analysis about AURKA expression and spliceosome activity score, r=0.57, p=0. **(b)** The correlation analysis about whole genome gene expression (clinical breast cancer samples from METABRIC) and spliceosome activity score. The correlation analysis about AURKA expression and spliceosome activity score, r=0.38, p=0.


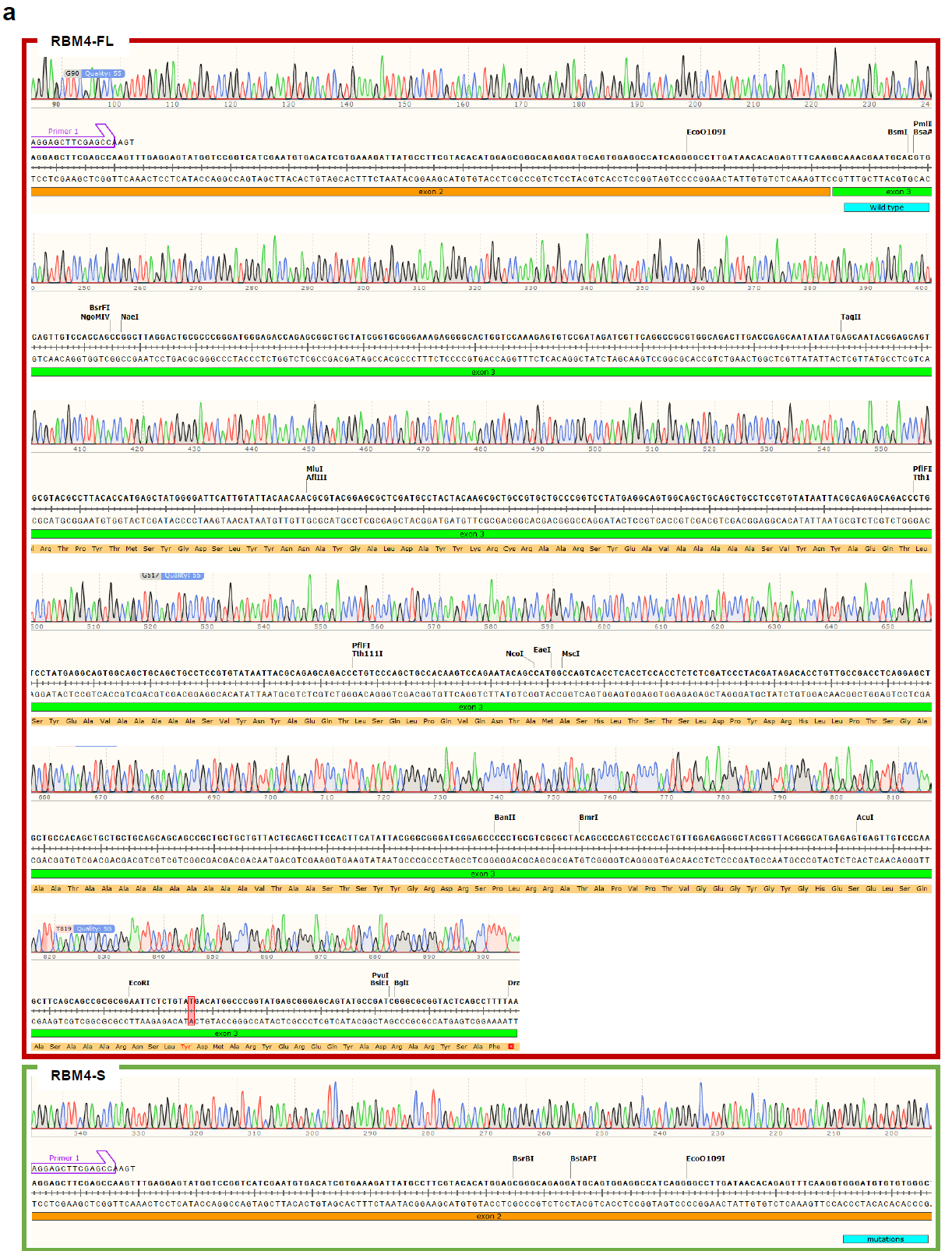


**Figure S12 Sequencing of purified PCR products of RBM4 two variants** **(a).**
