# Supplementary figures and images for: Nuclear Aurora kinase A switches m6A reader YTHDC1 to enhance an oncogenic RNA splicing of tumor suppressor RBM4
Source: Signal Transduct Target Ther. 2022 Apr 1;7:97. doi: 10.1038/s41392-022-00905-3 (PMC8971511; doi:10.1038/s41392-022-00905-3)

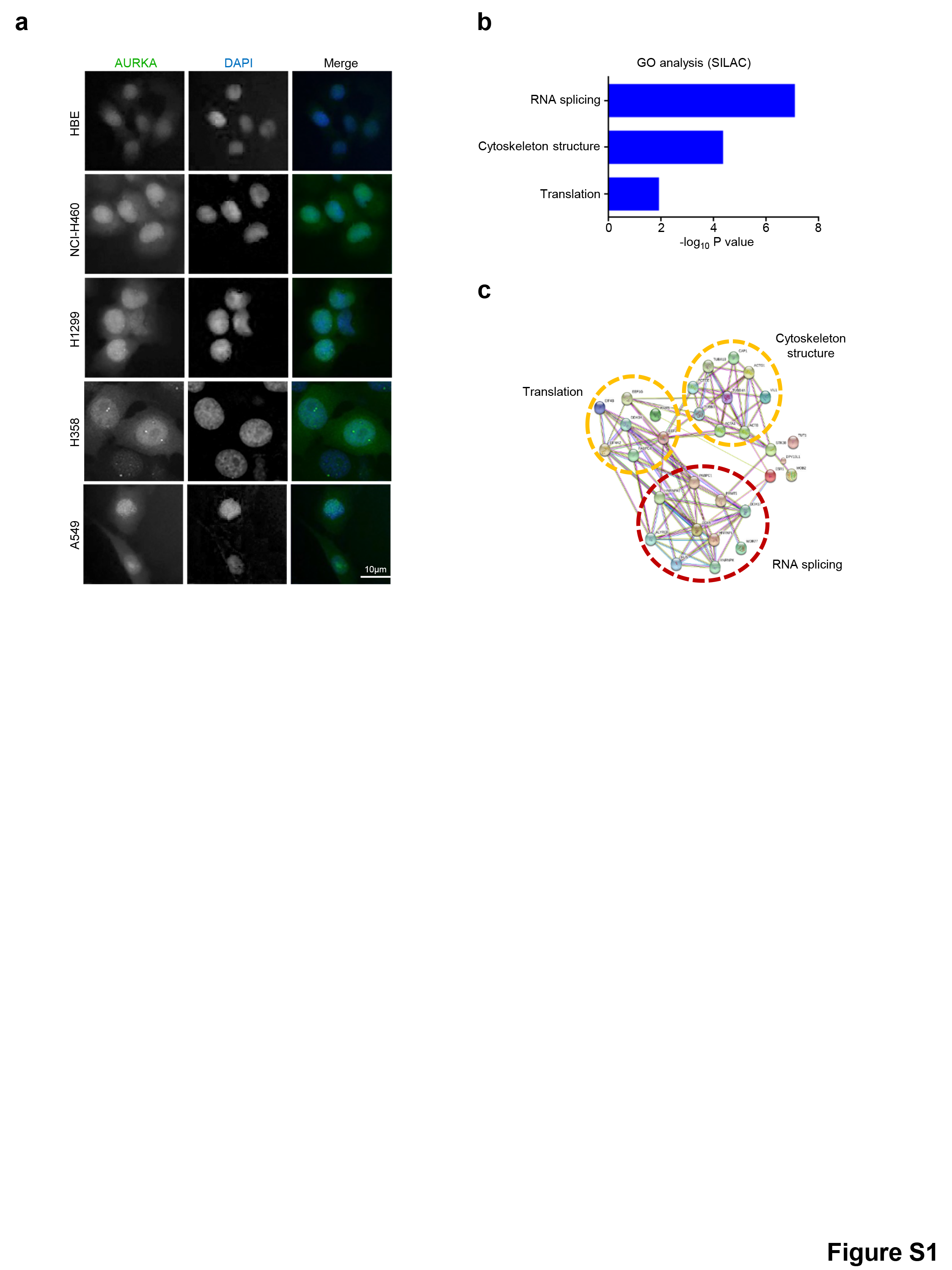

Supplement: Supplementary file 2 — Supplementary figure 1 [file 41392_2022_905_MOESM2_ESM.tif]

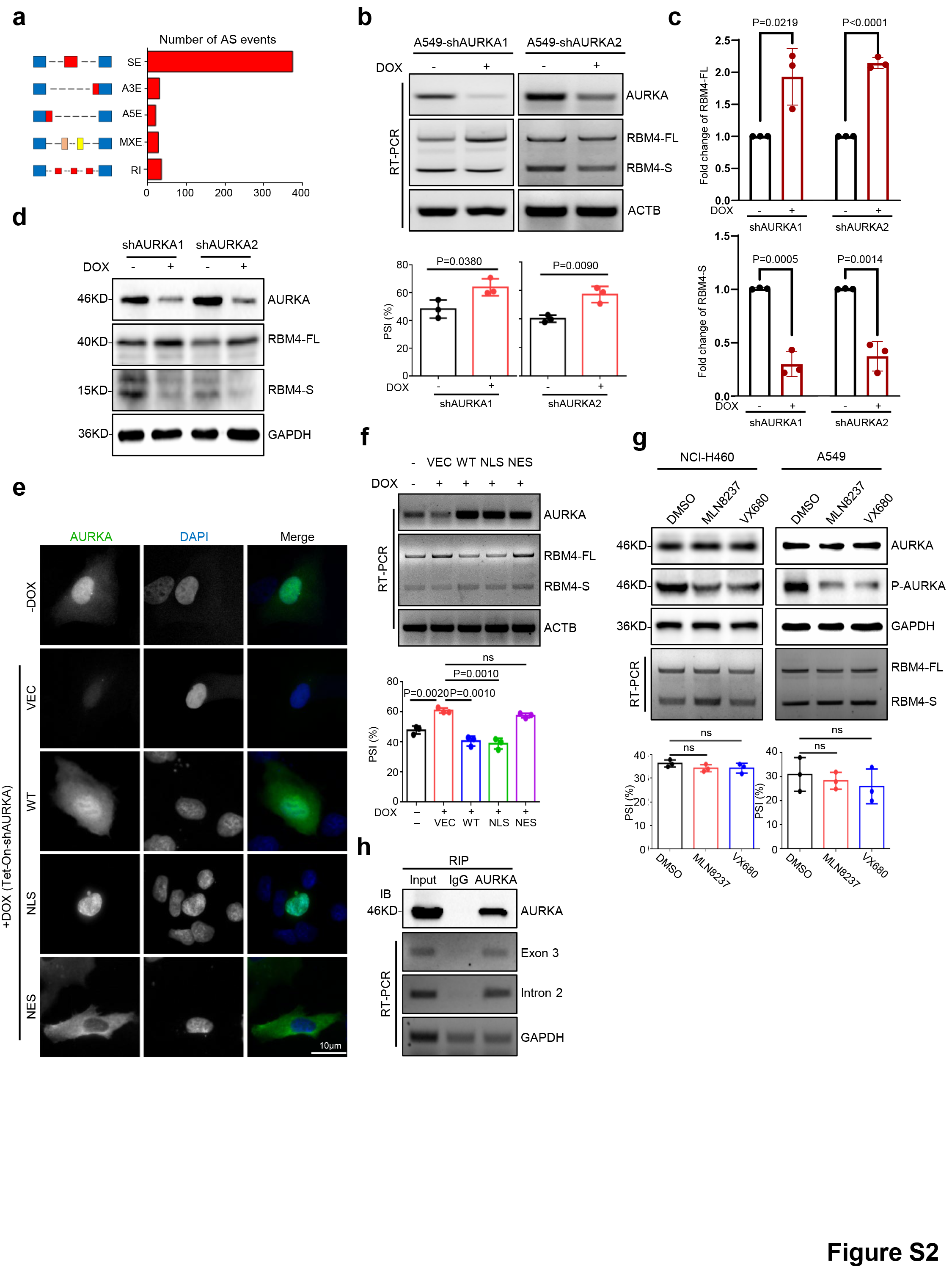

Supplement: Supplementary file 3 — Supplementary figure 2 [file 41392_2022_905_MOESM3_ESM.tif]

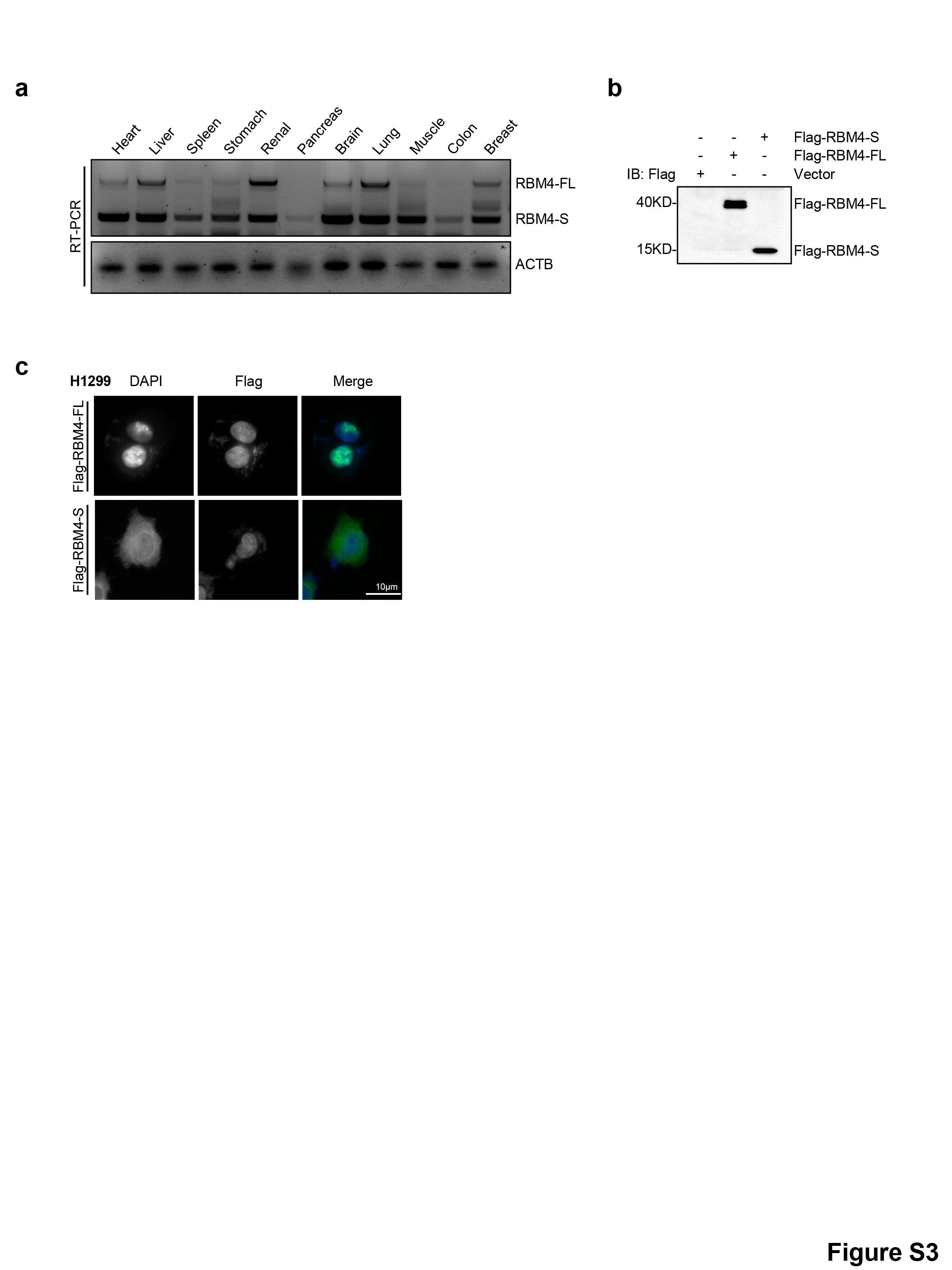

Supplement: Supplementary file 4 — Supplementary figure 3 [file 41392_2022_905_MOESM4_ESM.tif]

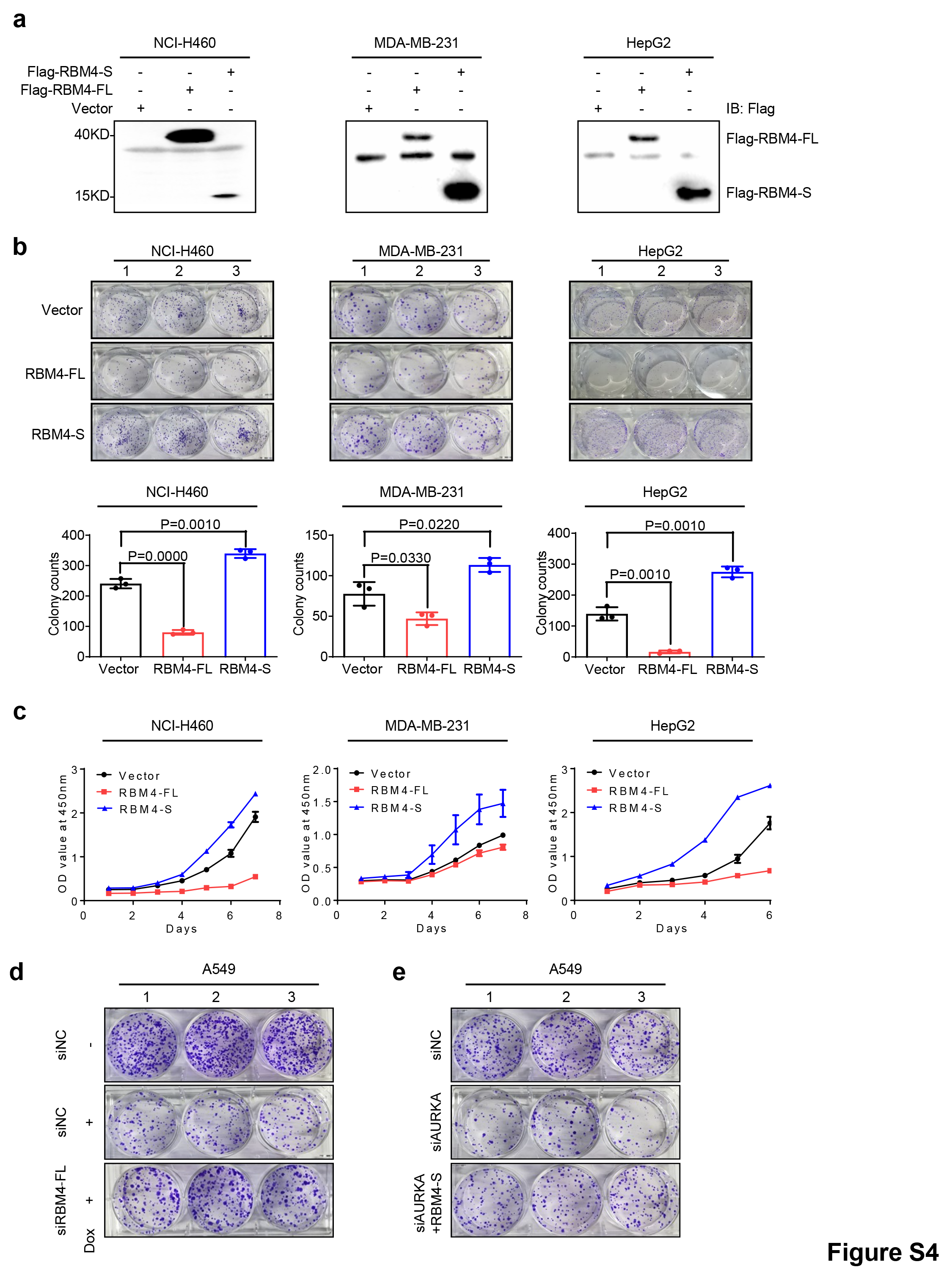

Supplement: Supplementary file 5 — Supplementary figure 4 [file 41392_2022_905_MOESM5_ESM.tif]

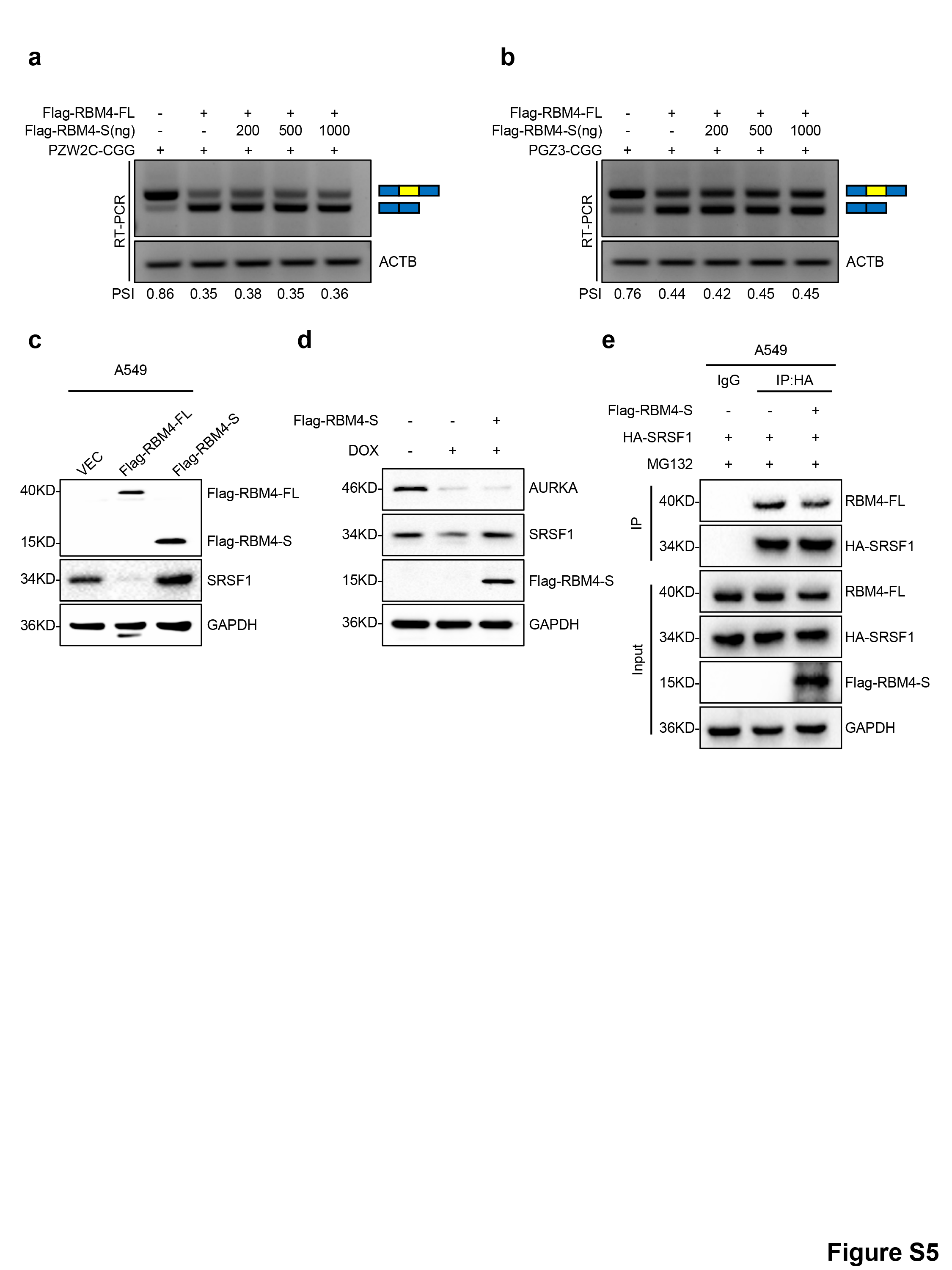

Supplement: Supplementary file 6 — Supplementary figure 5 [file 41392_2022_905_MOESM6_ESM.tif]

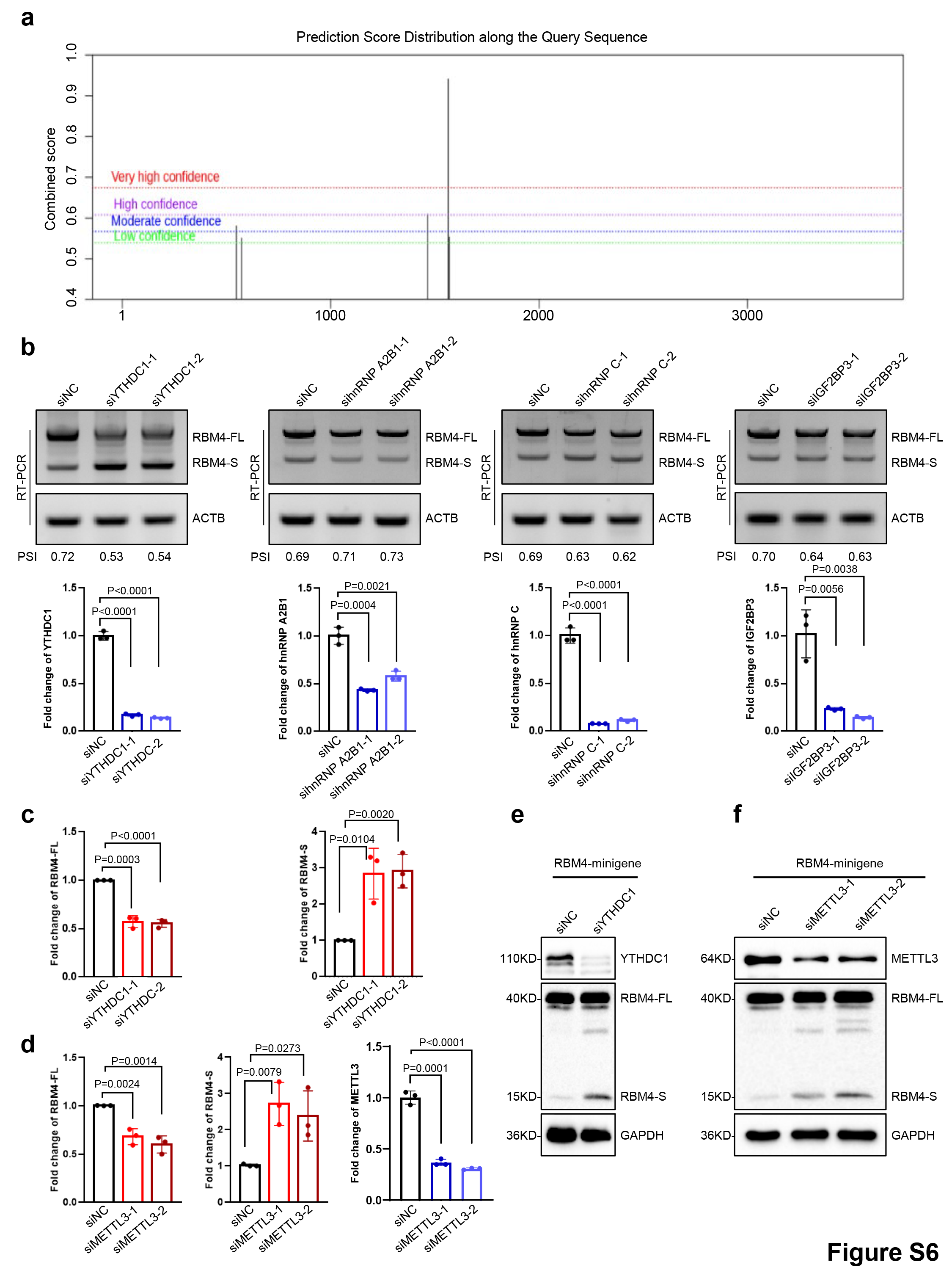

Supplement: Supplementary file 7 — Supplementary figure 6 [file 41392_2022_905_MOESM7_ESM.tif]

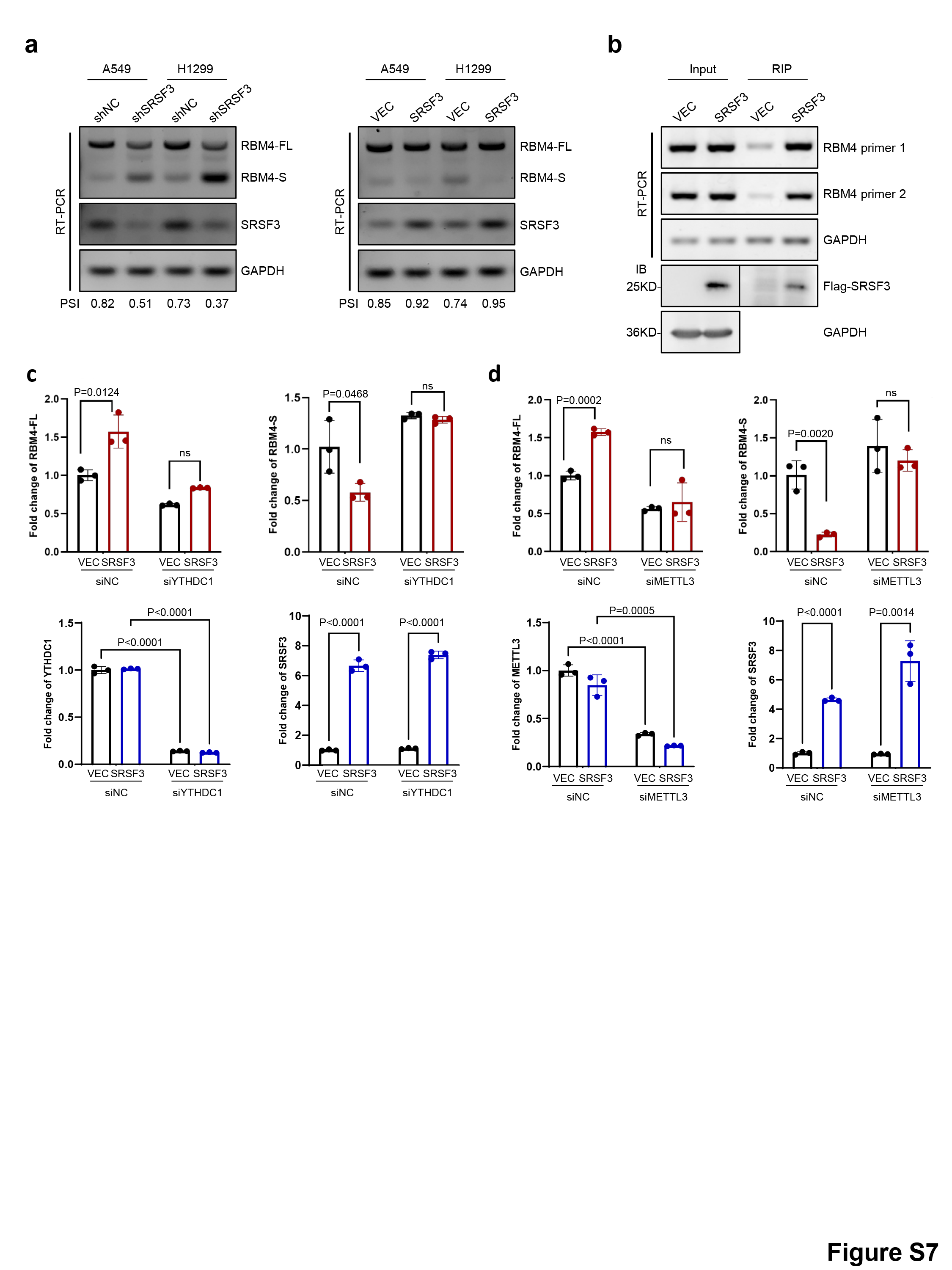

Supplement: Supplementary file 8 — Supplementary figure 7 [file 41392_2022_905_MOESM8_ESM.tif]

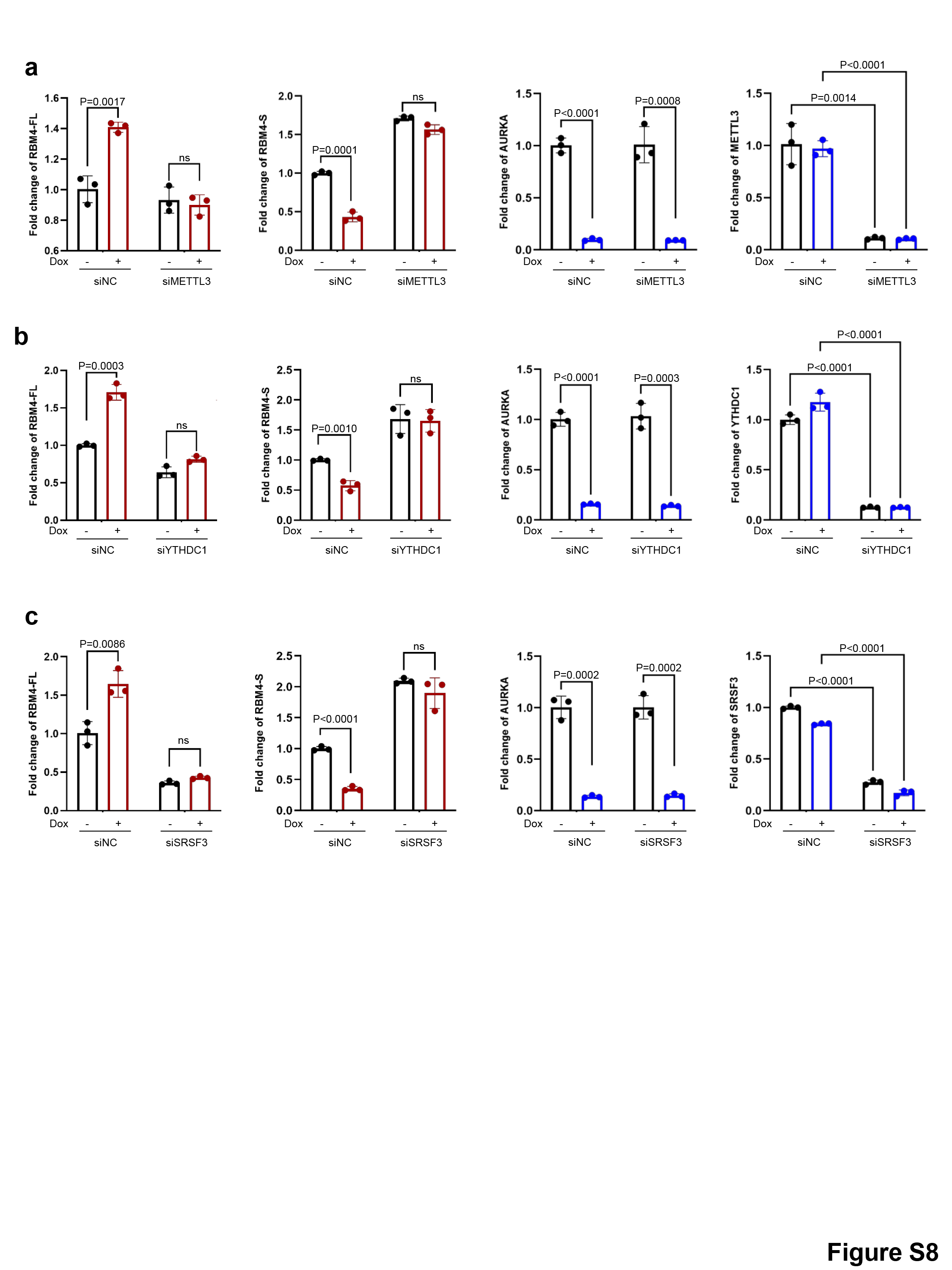

Supplement: Supplementary file 9 — Supplementary figure 8 [file 41392_2022_905_MOESM9_ESM.tif]

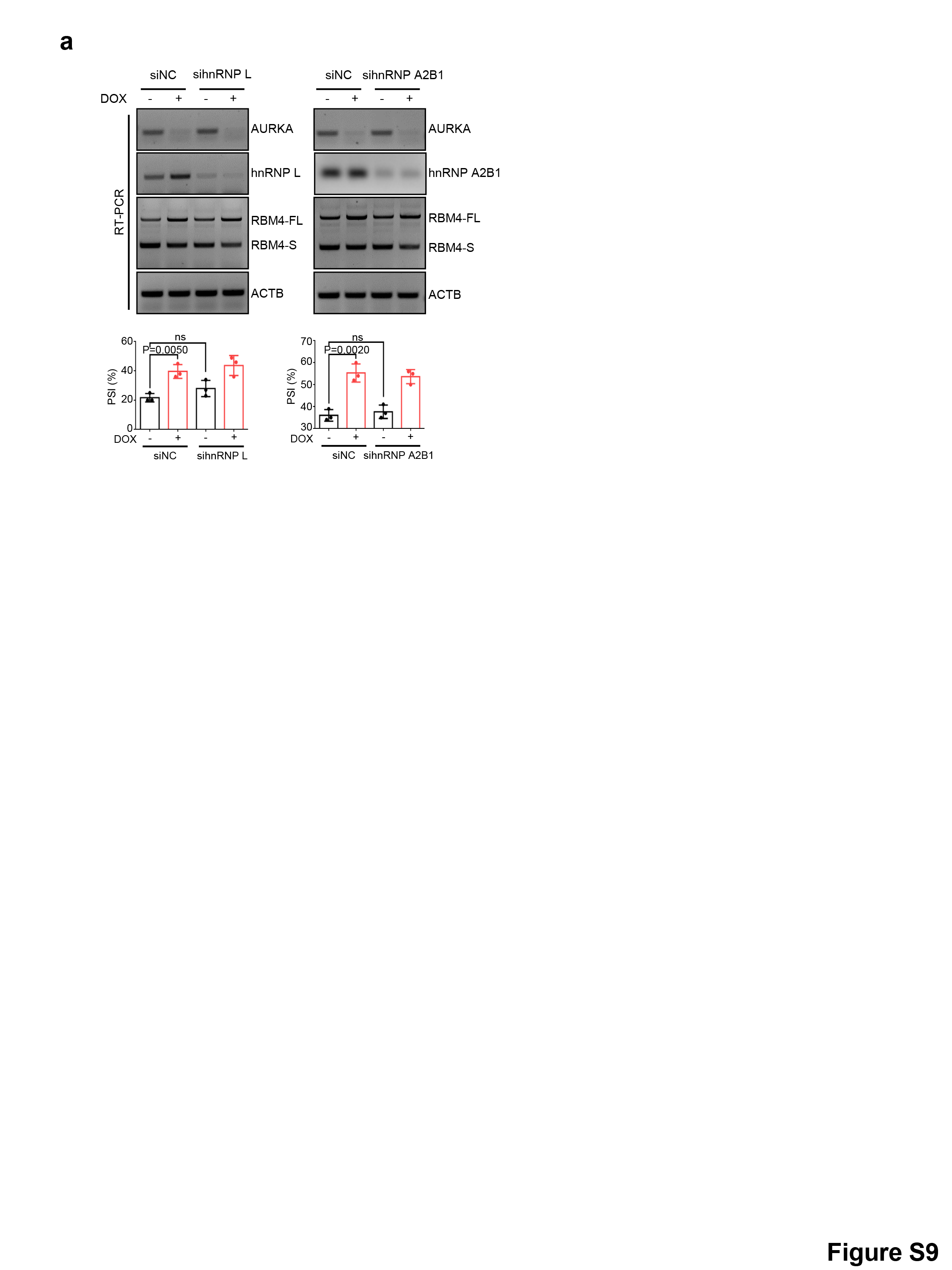

Supplement: Supplementary file 10 — Supplementary figure 9 [file 41392_2022_905_MOESM10_ESM.tif]

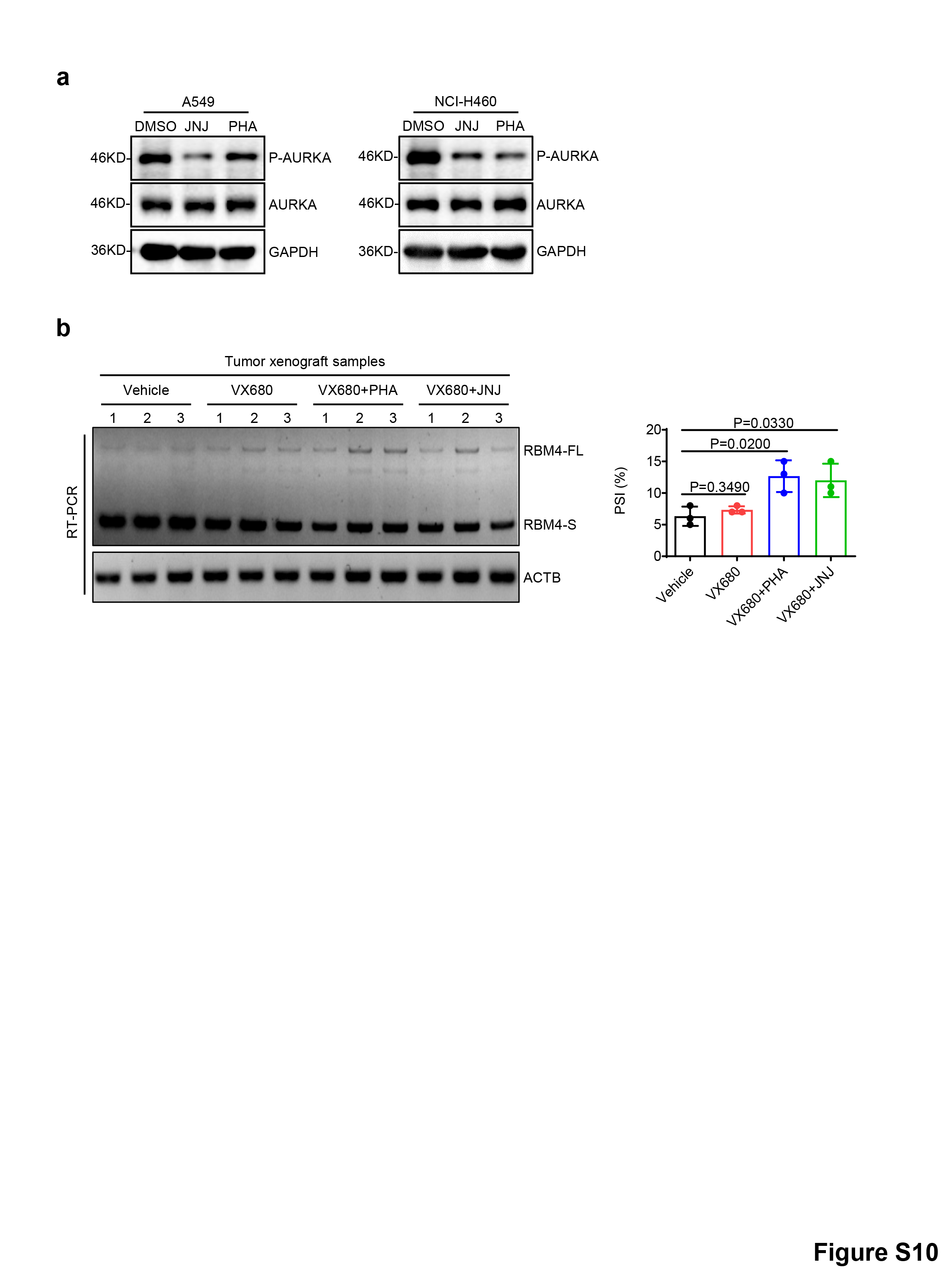

Supplement: Supplementary file 11 — Supplementary figure 10 [file 41392_2022_905_MOESM11_ESM.tif]

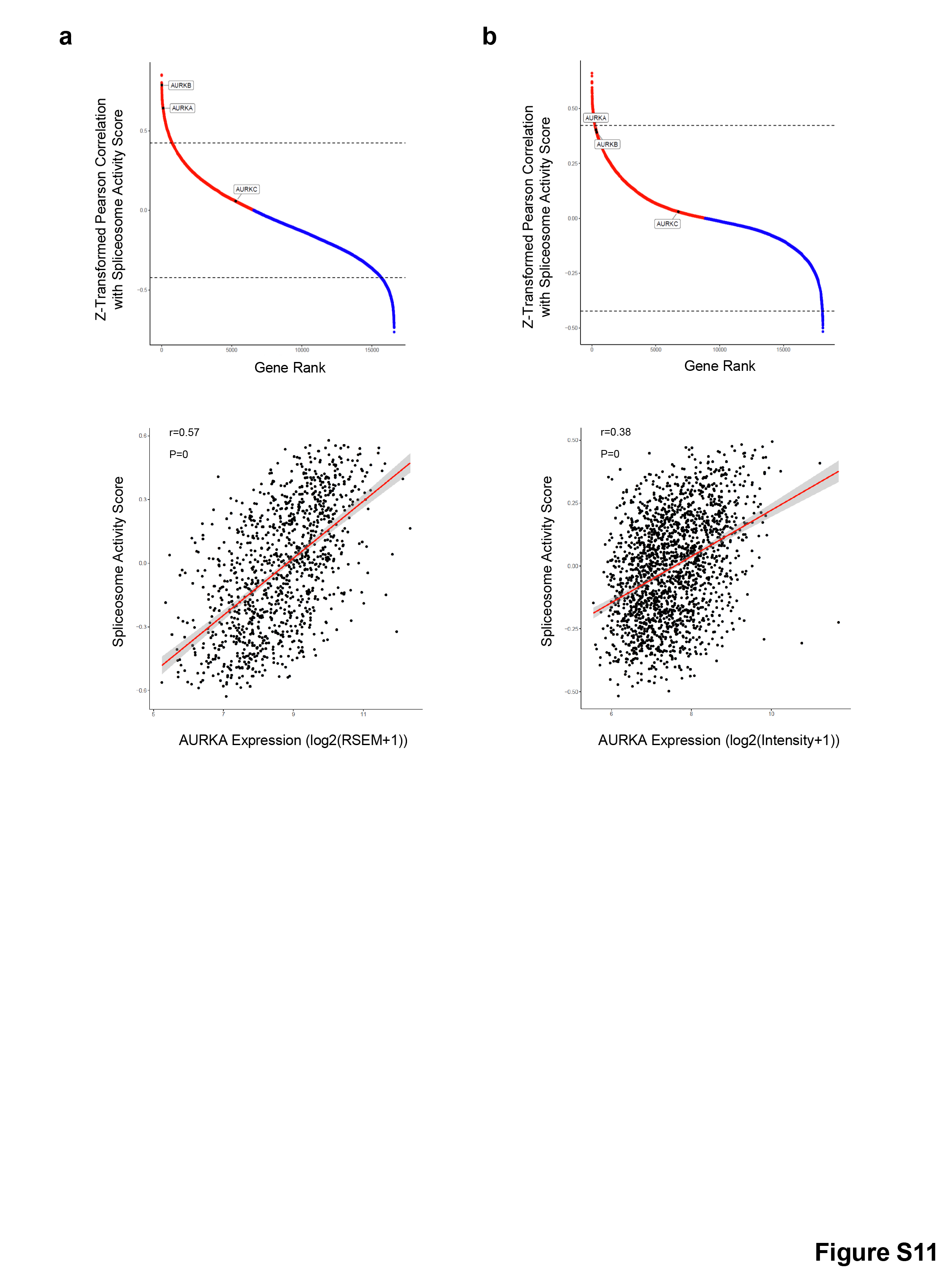

Supplement: Supplementary file 12 — Supplementary figure 11 [file 41392_2022_905_MOESM12_ESM.tif]

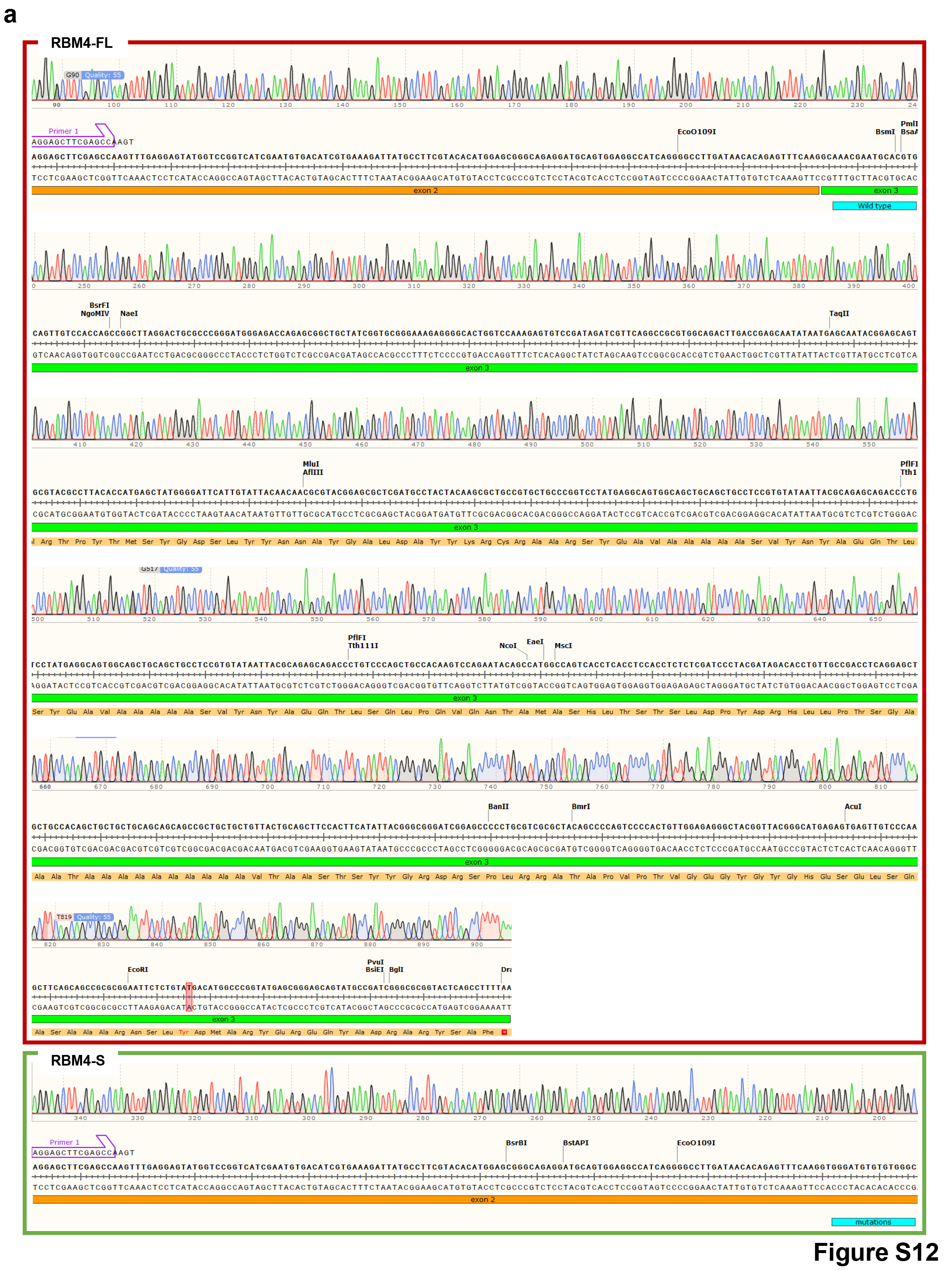

Supplement: Supplementary file 13 — Supplementary figure 12 [file 41392_2022_905_MOESM13_ESM.tif]
